# Supplementary material for: A library of polytypic copper-based quaternary sulfide nanocrystals enables efficient solar-to-hydrogen conversion
Source: Nat Commun. 2022 Sep 15;13:5414. doi: 10.1038/s41467-022-33065-7 (PMC9477825; doi:10.1038/s41467-022-33065-7)
Supplement: Supplementary file 1 — Supplementary Information [file 41467_2022_33065_MOESM1_ESM.pdf]

Supplementary Information

A library of polytypic copper-based quaternary sulfide nanocrystals enables efficient solar-to-hydrogen conversion

Wu et al.

`

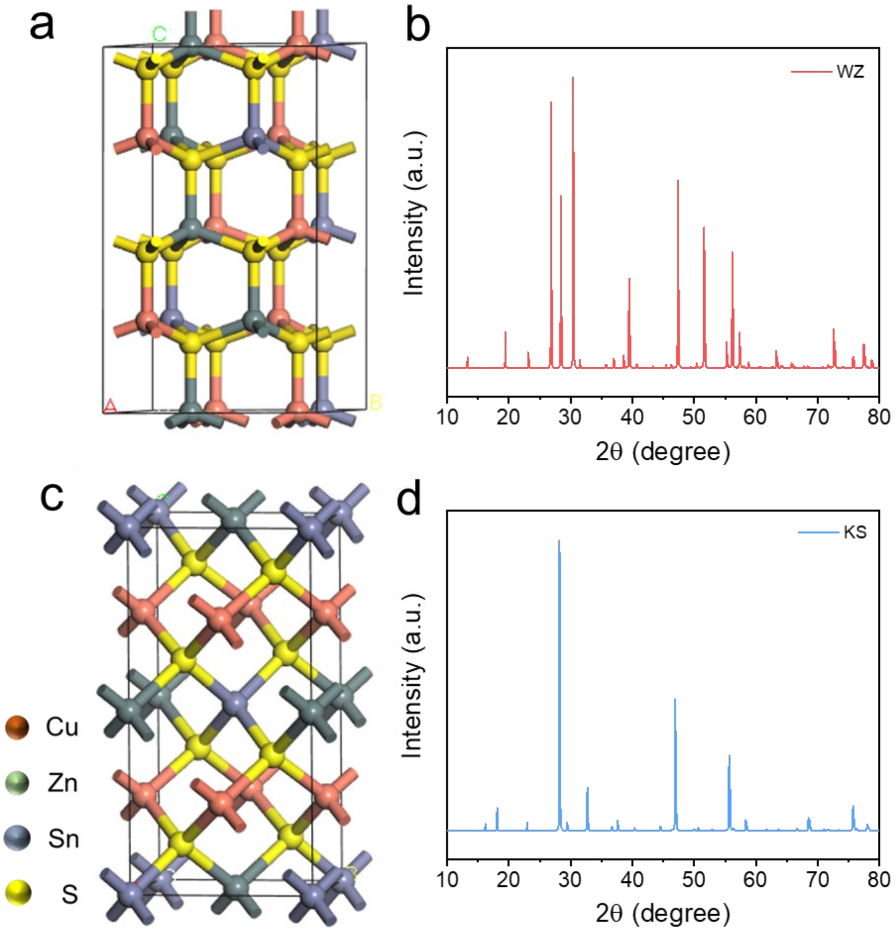

Supplementary Figure 1 | Structure models of CZTS as well as their XRD patterns, respectively. a-b, Wurtzite (WZ). **c-d**, Kesterite (KS).

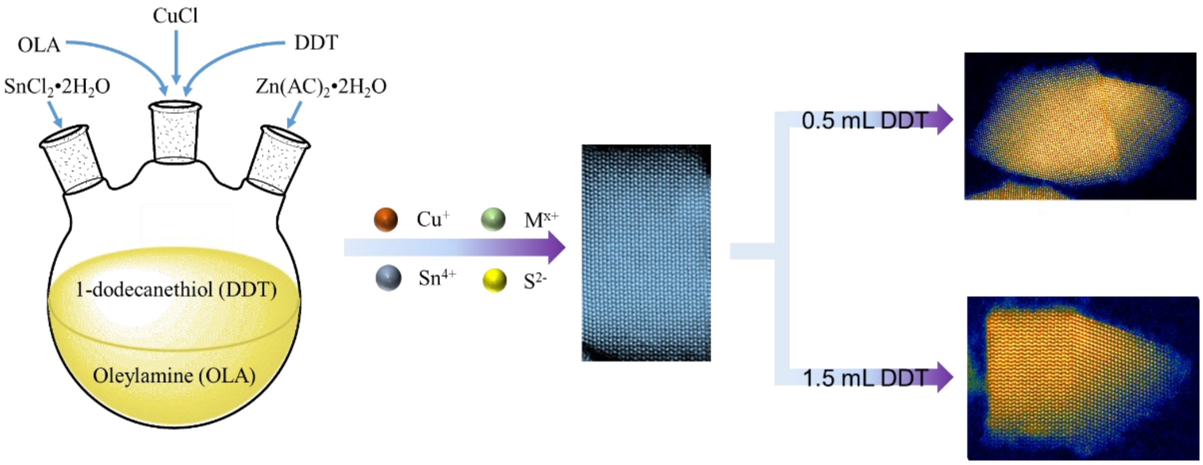

Supplementary Figure 2 | Scheme of the colloidal synthesis of polytypic CZTS nanocrystals. DHP CZTS nanocrystals would be obtained with 0.5 mL DDT and SHP CZTS nanocrystals could be synthesized with 1.5 mL DDT.

The formation mechanism of SHP CZTS nanocrystals has been investigated in detail. The time-dependent experiments have been conducted to reveal the growth process of SHP polytypic CZTS nanocrystals. First, the Cu_2S nanocrystals nucleate at low temperature (Supplementary Fig. 3a-c, Supplementary Fig. 4), followed by diffusion of Zn and Sn ions into Cu_2S nanocrystals to form wurtzite CZTS nanocrystals and nanocylinders (Supplementary Fig. 3d-g, Supplementary Fig. 4), and then KS CZS nucleates on the -(0001) facet of wurtzite CZTS to form a bullet-shaped polytypic nanocrystals with only one homojunctions (Supplementary Fig. 3h-k, Supplementary Fig. 4).

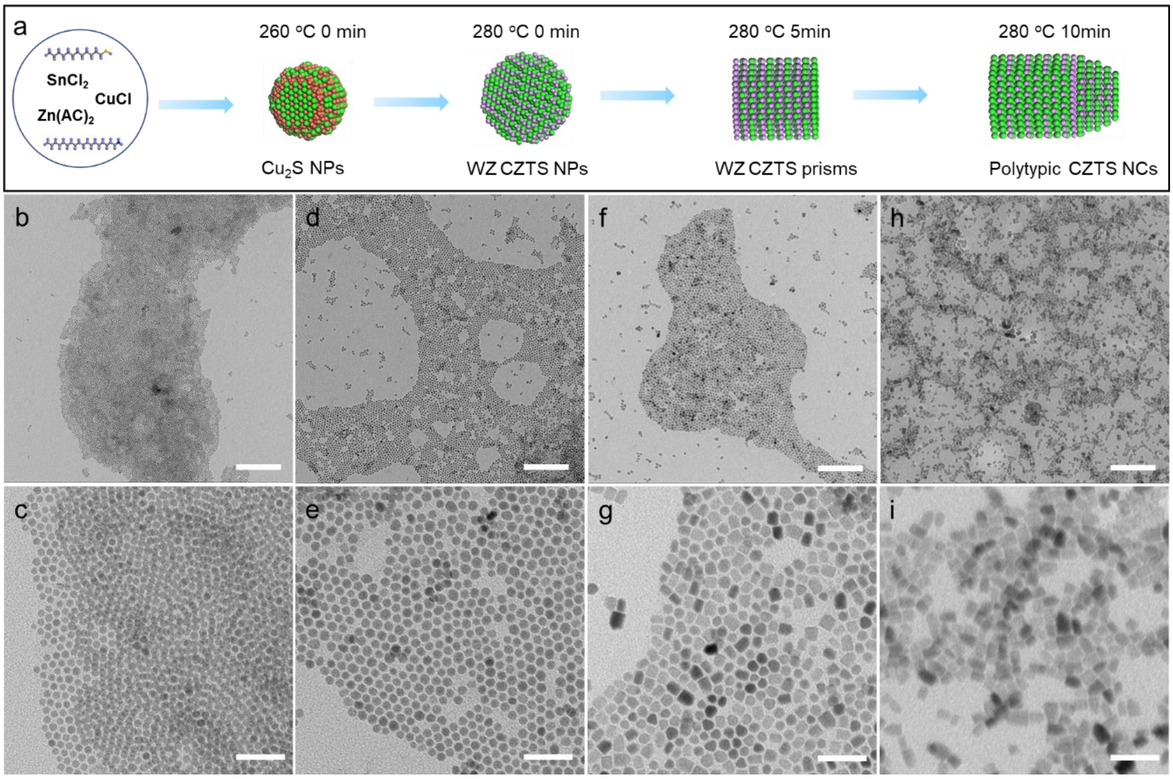

Supplementary Figure 3 | a, Schematic illustration of the growth mechanisms of SHP nanocrystals. **b-i,** TEM and HRTEM images of the nanocrystals obtained at different stages of the synthesis process of the SHP nanocrystals: **b-c,** The reaction temperature reached 260 °C. **d-e,** The reaction reached 280 °C. **f-g,** The reaction was kept at 280 °C for 5 min. **h-i,** The reaction was kept at 280 °C for 15 min. Scale bars are 200 nm for **b**, **d, f** and **h,** 50 nm for **c**, **e,** **g** and **i**, respectively.

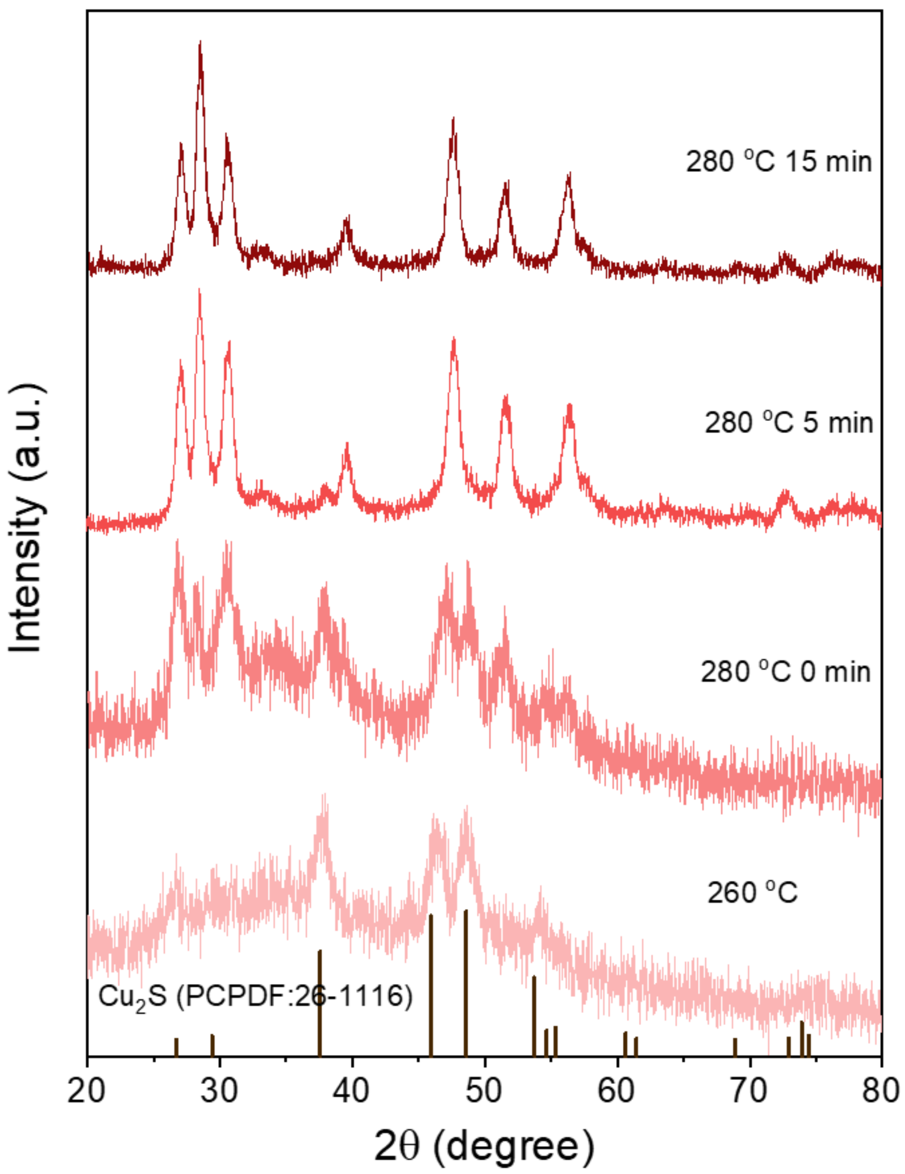

Supplementary Figure 4 | XRD patterns of the nanocrystals obtained at different stages of the synthesis process of the SHP nanocrystals.

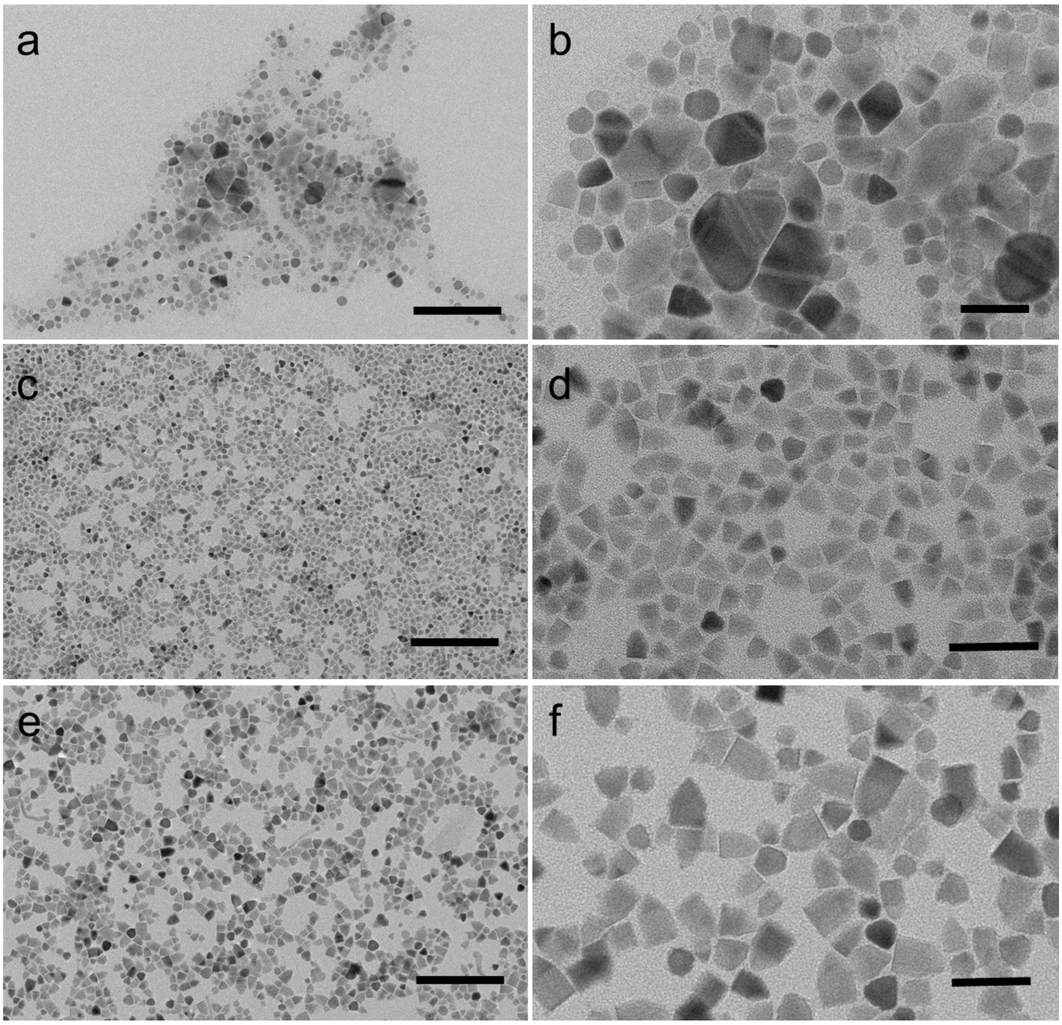

Supplementary Figure 5 | TEM images of the CZTS nanocrystals synthesized with different dosages of 1-DDT. **a-b**, 0.2 mL. The obtained CZTS nanocrystals have an irregular morphology. **c-d**, 1.0 mL. Both bullet-shaped and rugby-shaped nanocrystals have been obtained. **e-f**, 2.0 mL. All of the obtianed nanocrystals crystallized in a bullet-shape. These results demonstrate that the dosage of 1-DDT is a key matter to control the morphology of the obtained CZTS nanocrystals. Scale bars are 200 nm for **a**, **c** and **e,** 50 nm for **b**, **d** and **f**, respectively.

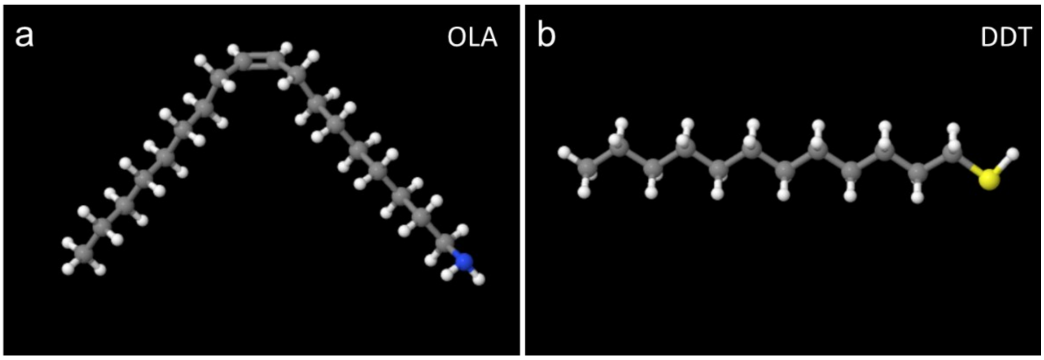

Supplementary Figure 6 | Structure models. **a**, OLA. **b**, DDT.

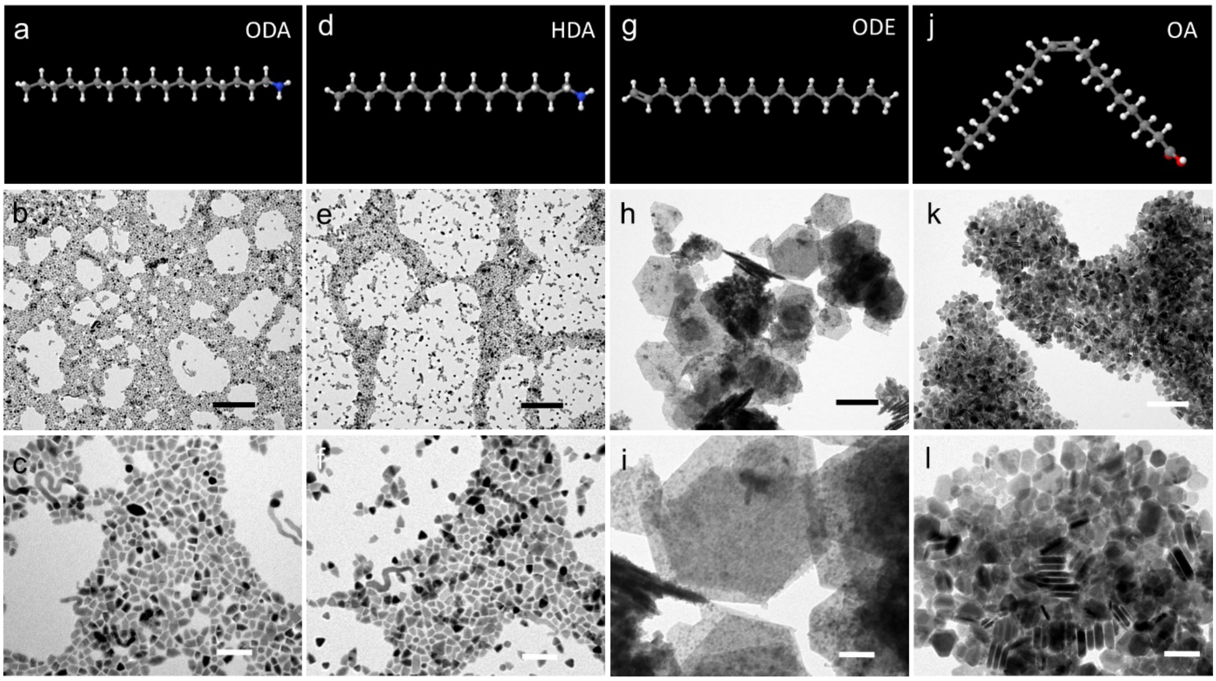

Supplementary Figure 7 | Characterization of the CZTS nanocrystals synthesized using different ligands. **a**, Structure of octadecylamine (ODA). **b-c**, TEM and magnified TEM images of the CZTS nanocrystals synthesized using ODA. **d**, Structure of 1-hexadecylamine (HAD). **e-f**, TEM and magnified TEM images of the CZTS nanocrystals synthesized using HDA. **g**, Structure of 1-octadecene (ODE). **h-i**, TEM and magnified TEM images of the CZTS nanocrystals synthesized using ODE. **j**, Structure of oleic acid (OA). **k-l**, TEM and magnified TEM images of the CZTS nanocrystals synthesized using OA. Scale bars are 200 nm for **b**, **e**, **h** and **k**, 50 nm for **c**, **f**, **i** and **l**, respectively.

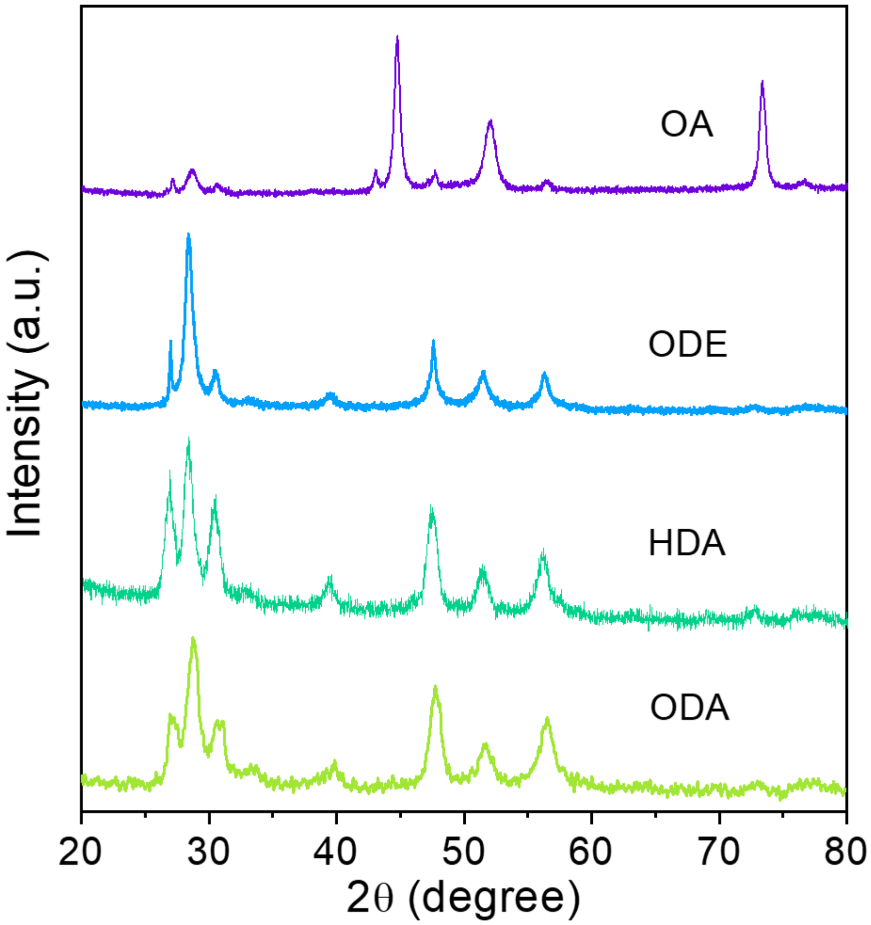

Supplementary Figure 8 | XRD patterns of CZTS nanocrystals synthesized using different ligands. As a result, polytypic CZTS nanocrystals will be obtained only when organic-amine is used as ligand and solvent.

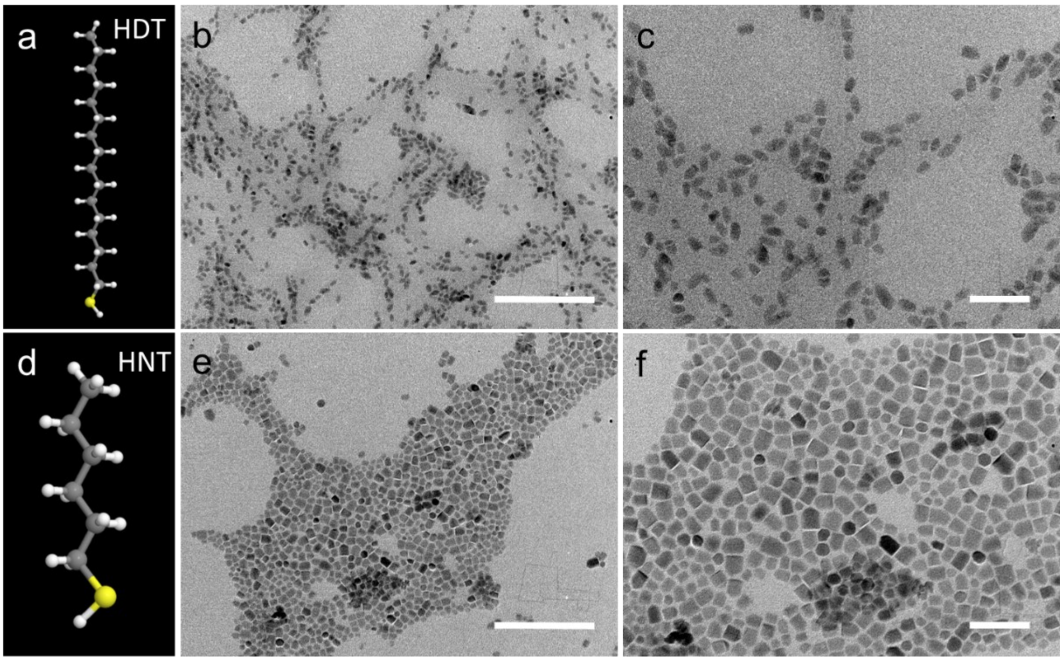

Supplementary Figure 9 | Characterization of the CZTS nanocrystals synthesized using different thiols. a, Structure of hexadecanethiol (HDT). **b-c**, TEM and magnified TEM images of the CZTS nanocrystals synthesized using HDT. **d**, Structure of hexanethiol (HNT). **e-f**, TEM and magnified TEM images of the CZTS nanocrystals synthesized using HNT. Scale bars are 200 nm for **b** and **e**, 50 nm for **c** and **f**, respectively.

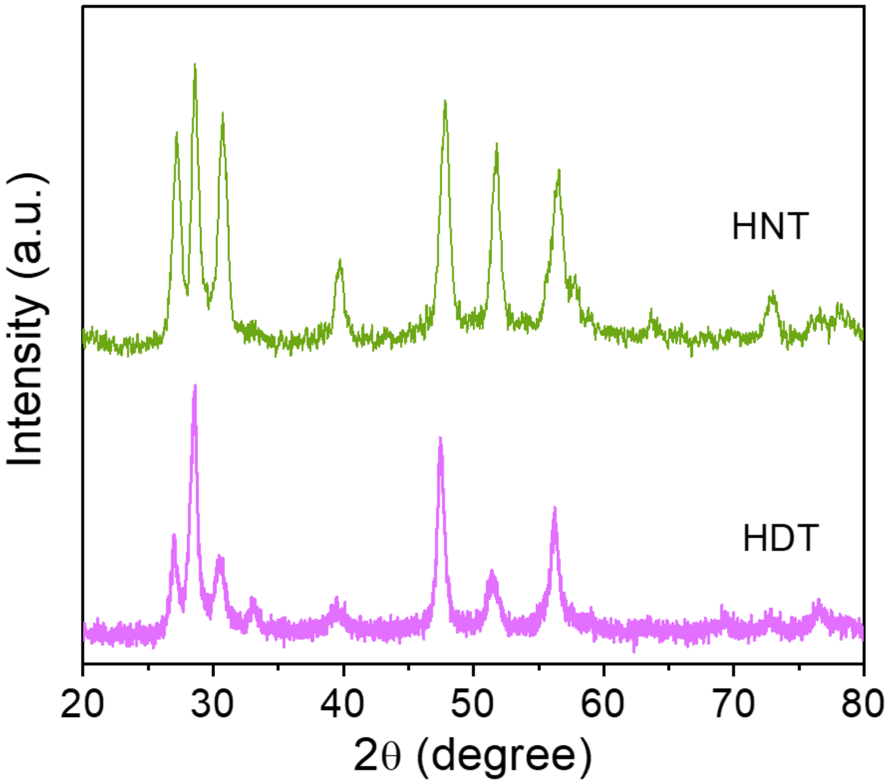

Supplementary Figure 10 | XRD patterns of the CZTS nanocrystals syntheiszed using different thiols. The TEM images (Supplementary Figure 6) and XRD patterns show that polytypic CZTS nanocrystals could be obtained when HDT and HNT were used. While, shorter alkyl chain leads to more ratio of wurtzite structure.

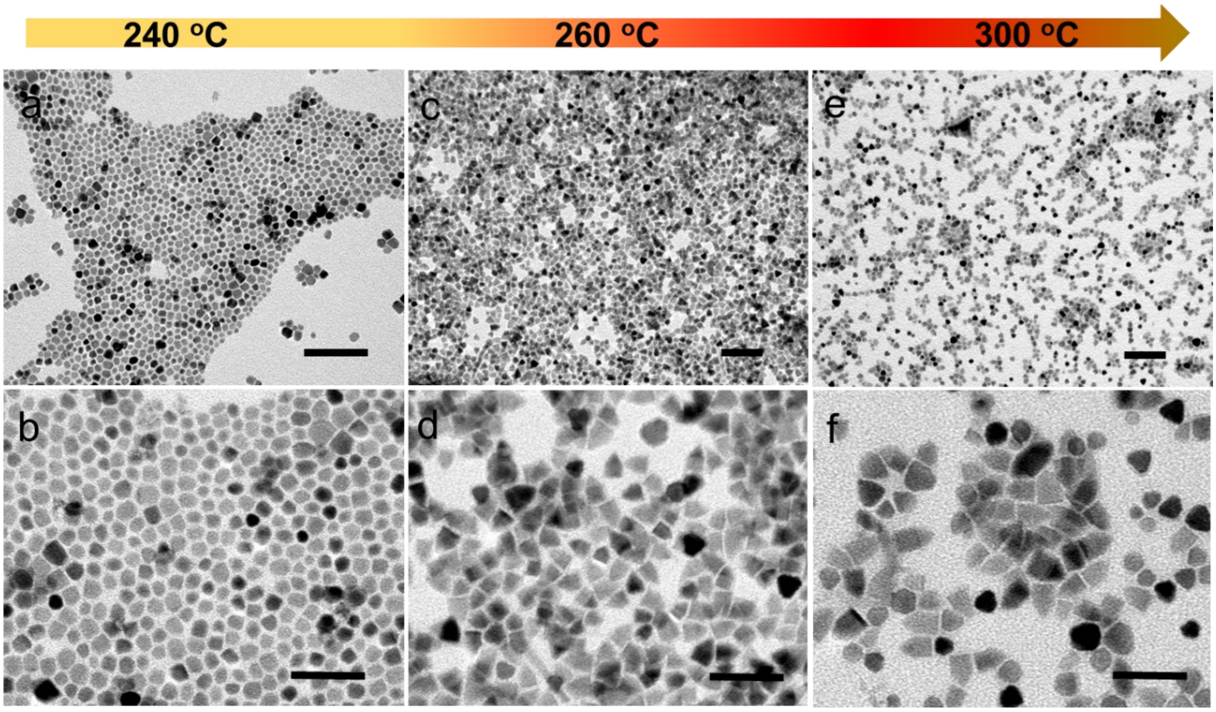

Supplementary Figure 11 |. TEM and magnified TEM images of CZTS nanocrystals syntheiszed at different temperature. **a-b**, 240 ºC. **c-d**, 260 ºC. **e-f**, 300 ºC. Scale bars are 100 nm for **a** and **c**, 200 nm for **e**, 50 nm for **b**, **d** and **f**, respectively.

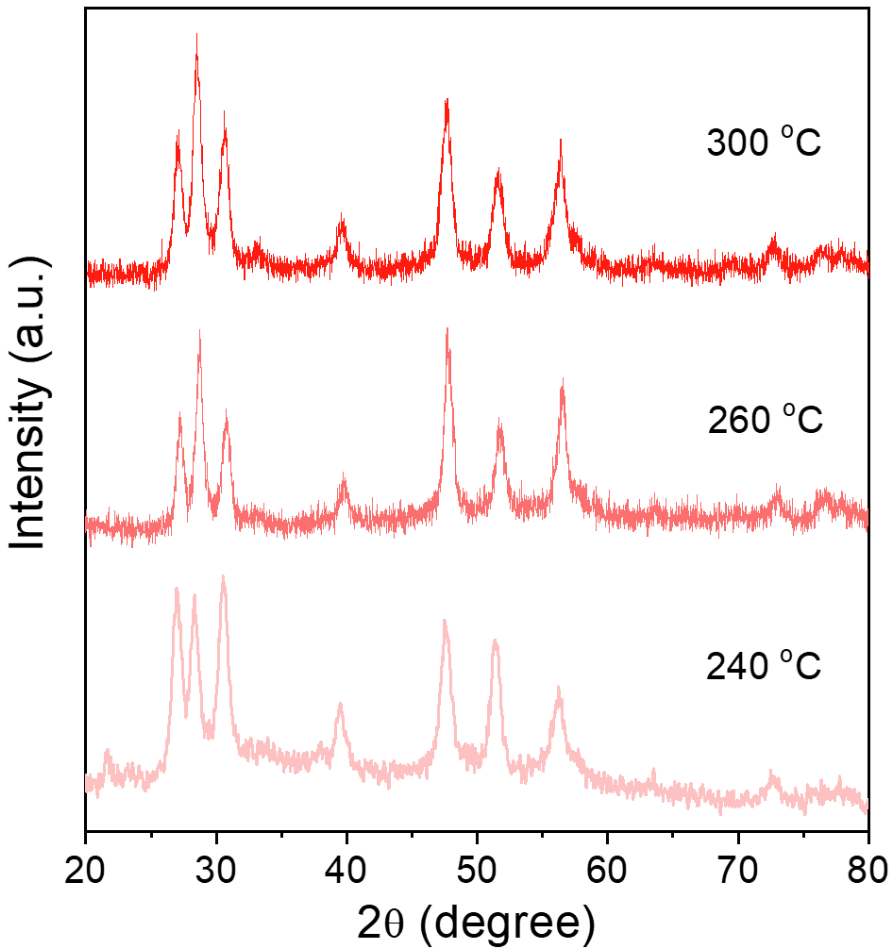

Supplementary Figure 12 | XRD patterns of the CZTS nanocrystals syntheiszed at different temperature. **a-b**, 240 °C. **c-d**, 260 °C. **e-f**, 300 °C. The results demonstrate that the reaction temperature is a key factor for synthesizing polytypic CZTS nanocrystals. Wurtzite CZTS nanocrystals are obtained at 240 °C, while, irregular CZTS nanocrystals will appear at 300 °C.

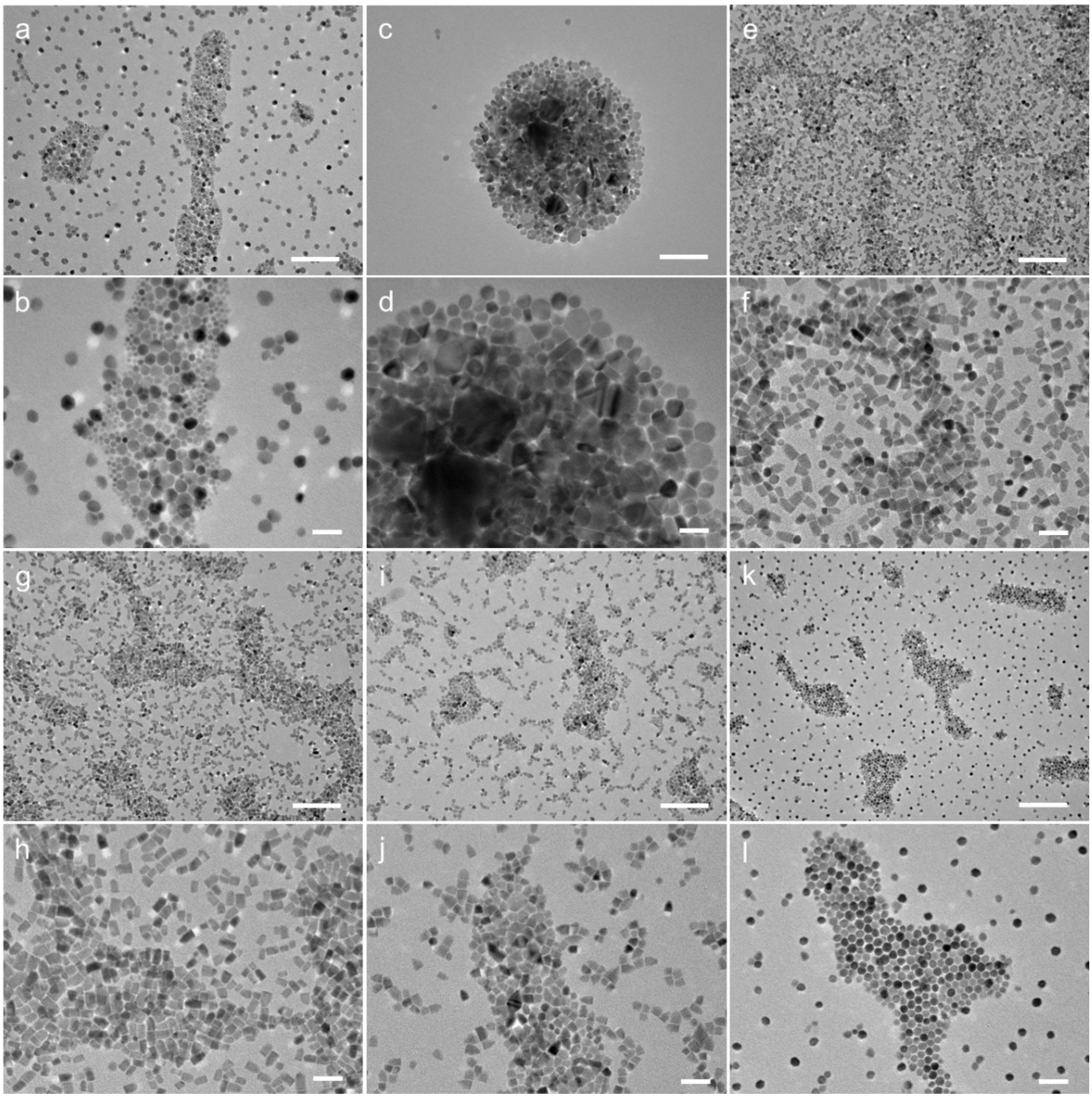

Supplementary Figure 13 |. TEM and magnified TEM images of polytypic CZTS nanocrystals synthesized with different Cu sources. **a-b**, $CuCl_2$, 0.5 mL of 1-DDT. **c-d**, $CuCl_2$, 1.5 mL of 1-DDT. **e-f**, $Cu(acac)_2$, 0.5 mL of 1-DDT. **g-h**, $Cu(acac)_2$, 1.5 mL of 1-DDT. **i-j**, $Cu(AC)_2$, 0.5 mL of 1-DDT. **k-l**, $Cu(AC)_2$, 1.5 mL of 1-DDT. Scale bars are 200 nm for **a, c**, **e, g, i,** and **k**, 50 nm for **b**, **d, f**, **h, j** and **l,** respectively.

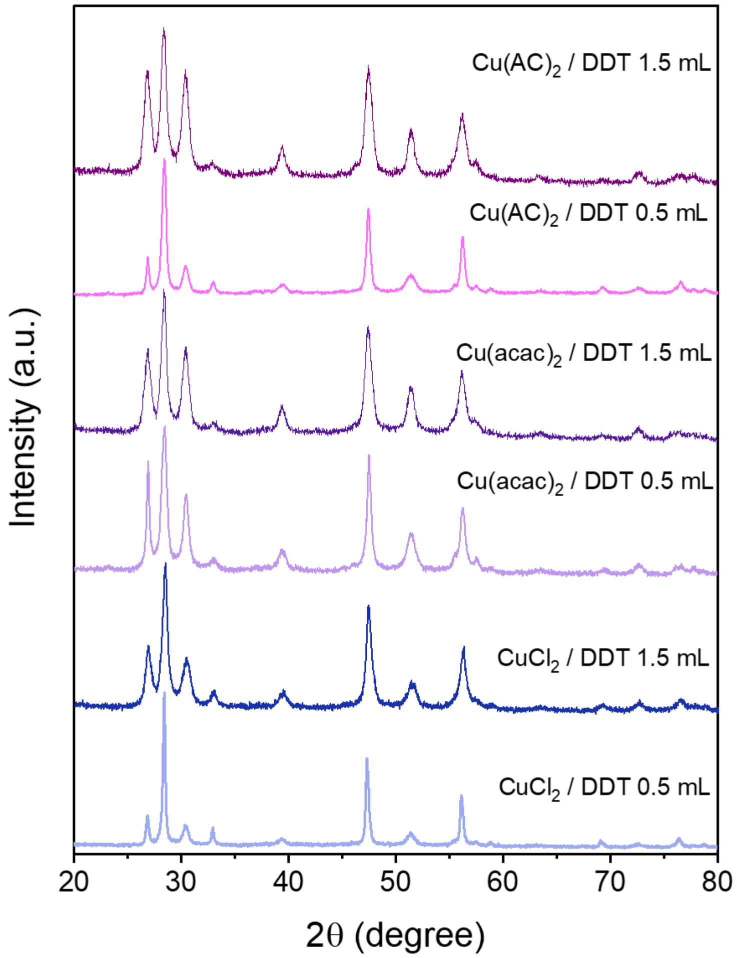

Supplementary Figure 14 |. XRD patterns of polytypic CZTS nanocrystals syntheiszed with differernt Cu sources.

When $CuCl_2 \cdot 2H_2O$ was used for synthesis of SHP and DHP CZTS nanocrystals, the irregular shape CZTS nanocrystals would be obtained (Supplementary Fig. 13a-d, Supplementary Fig. 14). CZTS nanorods would be synthesized by using $Cu(acac)_2$ as Cu precursor (Supplementary Fig. 13e-h, Supplementary Fig. 14). When $Cu(AC)_2$ was used as Cu source, bullet-shape polytypic CZTS nanocrystals were obtained with 0.5 mL of 1-DDT and CZTS nanoplates were produced with 1.5 mL of 1-DDT (Supplementary Fig. 13i-l, Supplementary Fig. 14). In conclusion, well-defined polytypic CZTS nanocrystals would be synthesized using specific Cu(II) precursors.

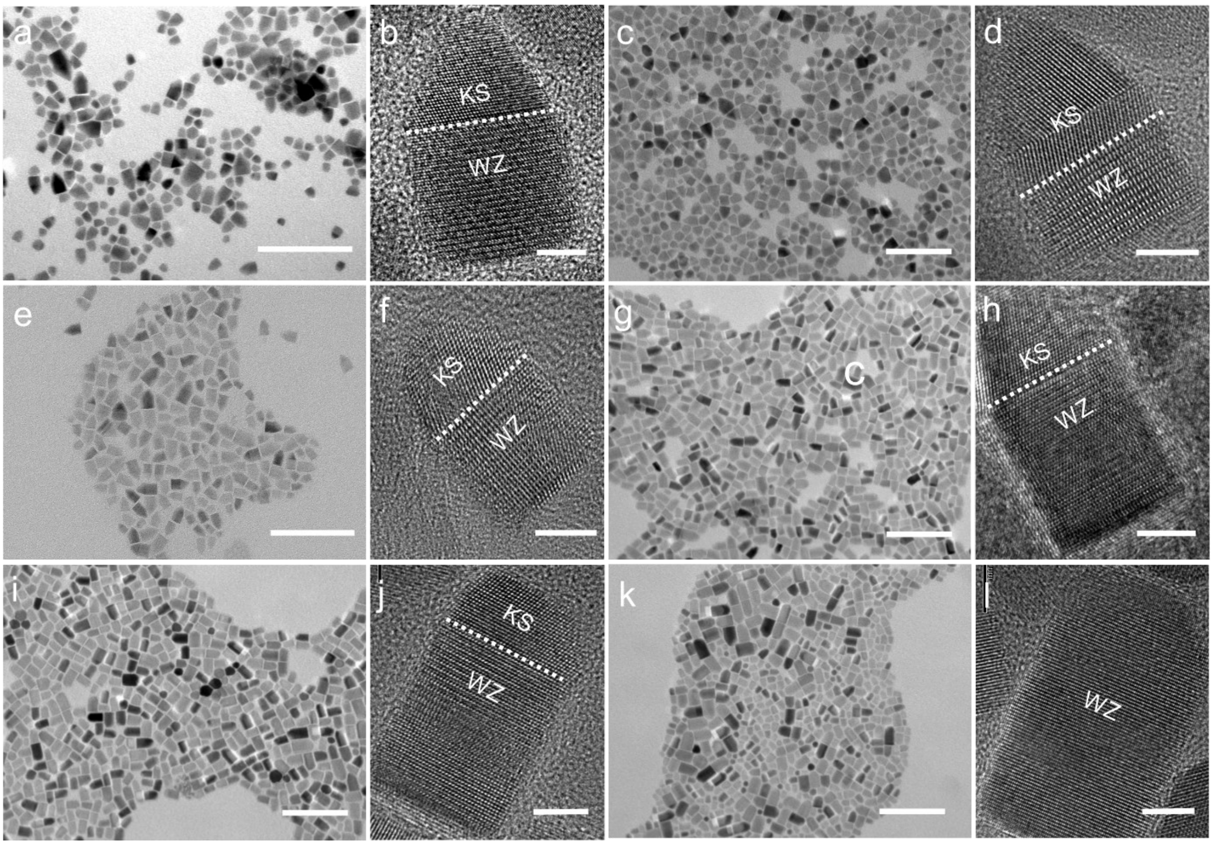

Supplementary Figure 15 | TEM and HRTEM images of the CZTS nanocrystals synthesized with different dosages of Zn(AC)$_2$·2H$_2$O. **a-b**, Z0, 0 mmol. **c-d**, Z1, 0.07 mmol. **e-f**, Z2, 0.14 mmol. **g-h**, Z4, 0.28 mmol. **i-j**, Z5, 0.35 mmol. **k-l**, Z6, 0.42 mmol. Experiments with different dosages of Zn(AC)$_2$ was taken to study the influence of Zn content on the morphology and structure of the obtained CZTS nanocrystals. The ratio of wurtzite structure increased with Zn content. When the dosage of Zn(AC)$_2$ arrived at 0.42 mmol, the obtained CZTS nanocrystals crystallized in a pure wurtzite structure. Scale bars are 100 nm for **a**, **c**, **e**, **g**, **i** and **k**, 5 nm for **b**, **d**, **f**, **h**, **j** and **l**, respectively.

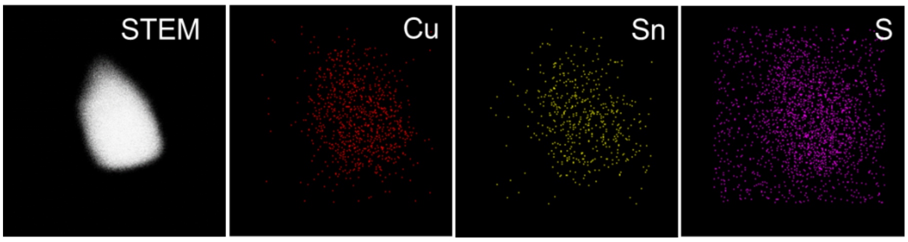

Supplementary Figure 16 | EDS element mapping of polytypic Cu_2SnS_3 (CTS) nanocrystals. The EDS element mapping shows the homogeneous distribution of Cu, Sn, and S elements.

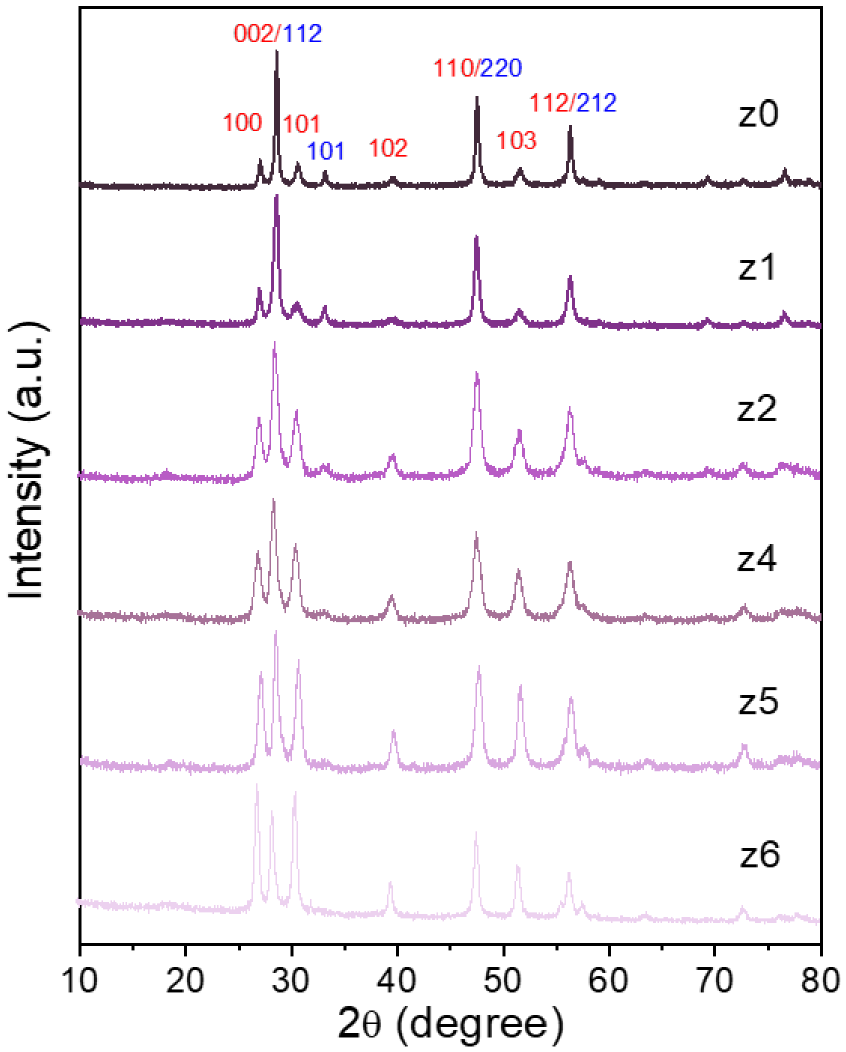

Supplementary Figure 17 | XRD patterns of CZTS nanocrystals synthesized with different dosages of $Zn(AC)_2$. The intence of 002/112 peaks decreased and the 101 peak of kesterite structure disappeared with increasing of Zn content. This result shows that the Zn content in the obtained CZTS nanocrystals can directly influnce their morpholoy and structure.

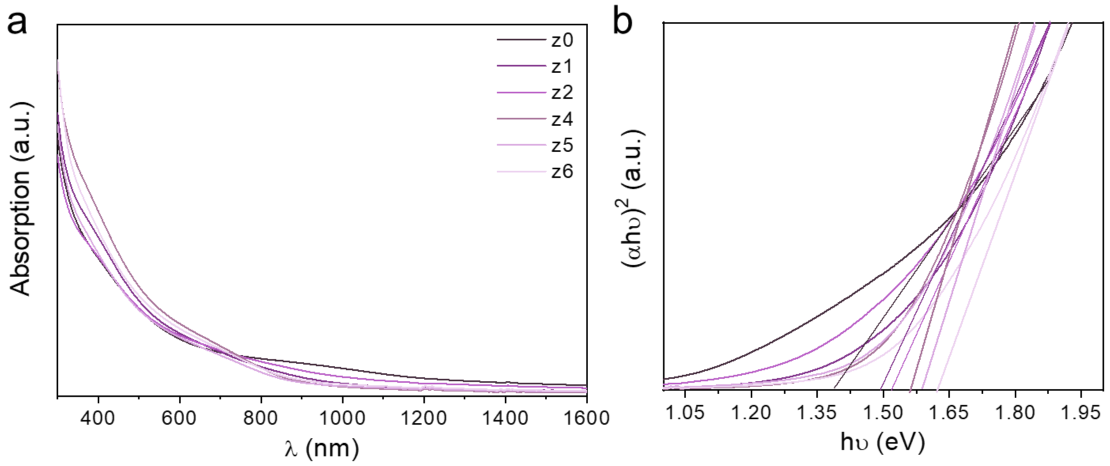

Supplementary Figure 18 | Optical properties of the CZTS nanocrystals synthesized with different dosages of Zn(AC)$_2$. a, UV-Vis-IR absorption properties of the CZTS nanocrystals synthesized with different amouts of Zn(AC)$_2$. **b**, The graph of linear extrapolation of $(\alpha h\upsilon)^2$ versus photon energy (hυ). The detail band gap informations were listed in Supplementary Table 1.

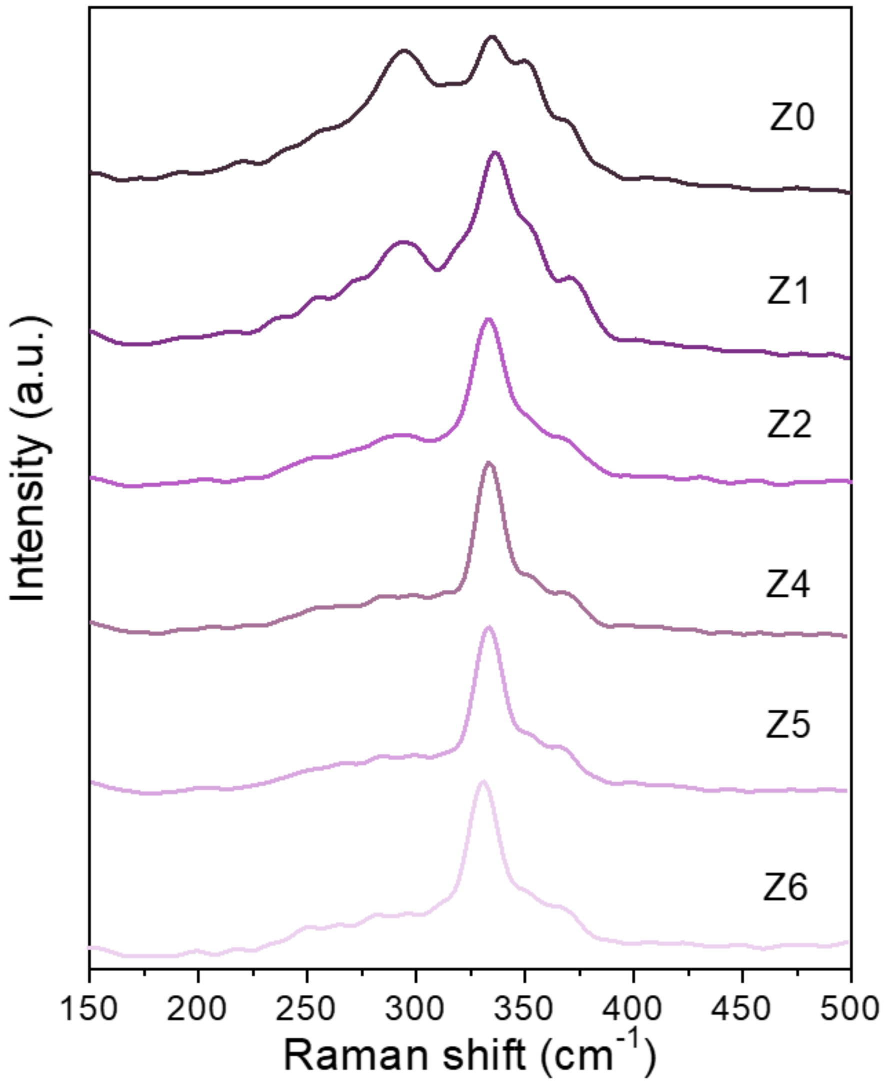

Supplementary Figure 19 | Raman spectra of the CZTS nanocrystals with different dosages of $Zn(AC)_2$. The results show that there is no exsit of binary ZnS with increasing Zn content.

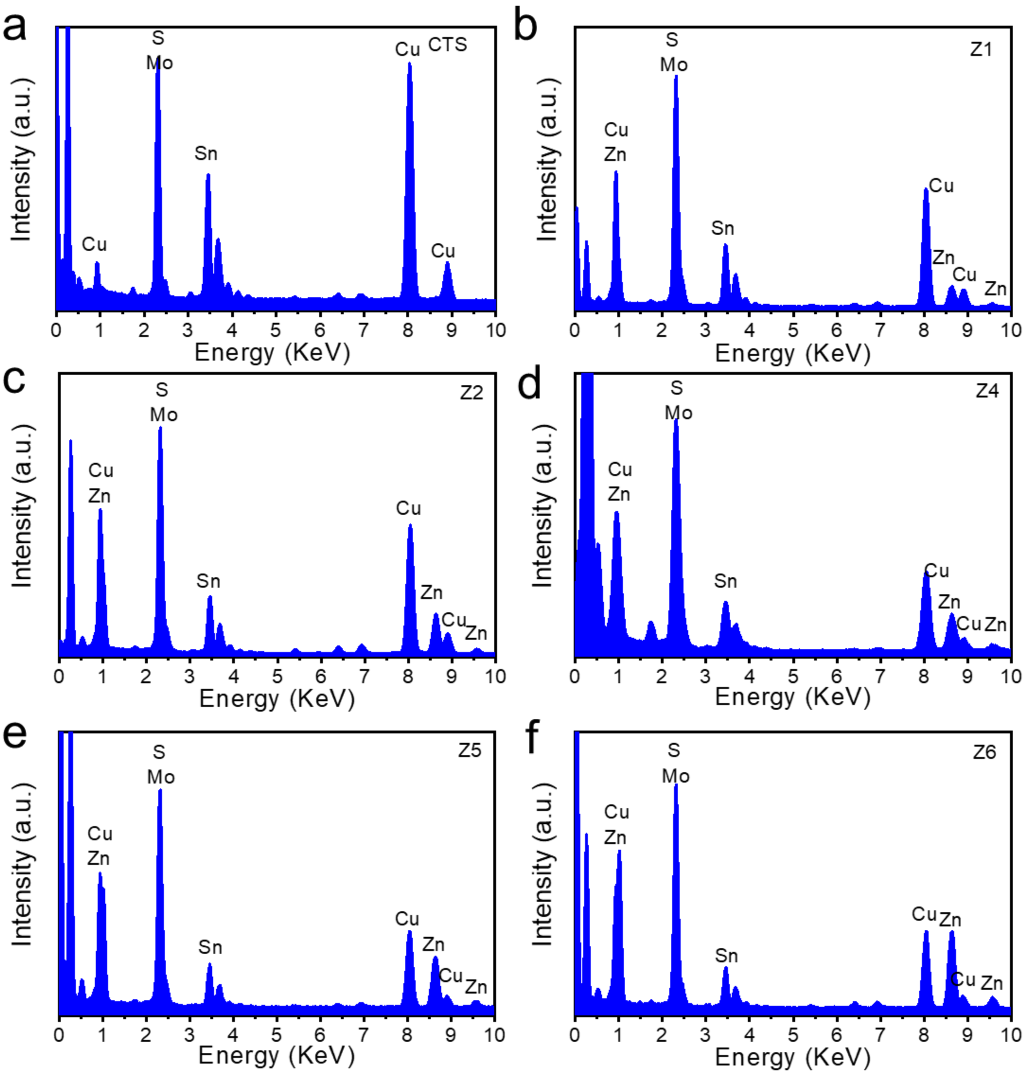

Supplementary Figure 20 | EDS spectra of the obtained CZTS nanocrystals synthesized with different amounts of $Zn(AC)_2$. a, Polytypic CTS. **b-f,** Polytypic CZTS nanocrystals with different contents of Zn. The detail cation ratios was listed in Supplementary Table 1.

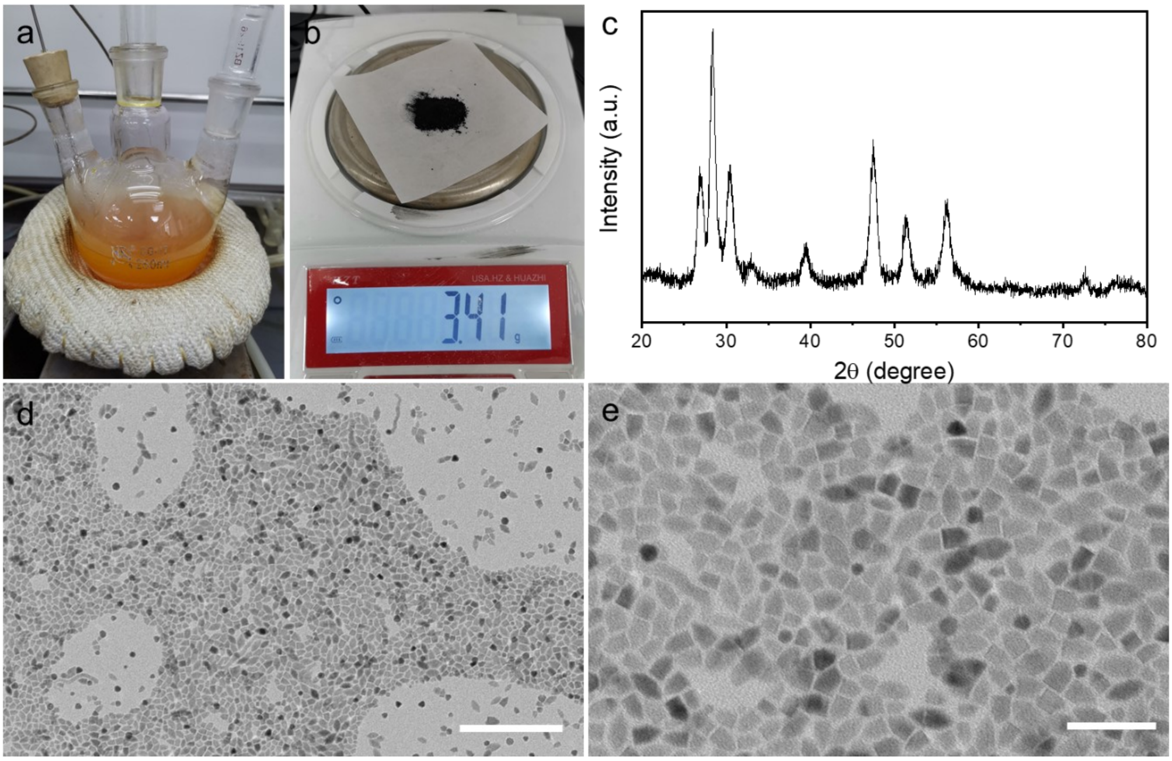

Supplementary Figure 21 | Characterization of the large-scale synthesized SHP nanocrystals. a, The photograph of the reaction solution color at 180 ºC. **b,** Photograph of 3.41 g of SHP nanocrystals powder. **c,** XRD patterns. **d-e,** TEM images. Scale bars are 200 nm for **a** 50 nm for **b**, respectively.

Hence, we try to large scale synthesis of SHP CZTS nanocrystals in a mix solution with 100 mL of OLA and 20 mL of 1-DDT. Supplementary Fig. 21a shows the reaction flask with the capacity of 250 mL contains metal cation precursors dissolved in 100 mL of OLA and 20 mL of 1-DDT. The photographic (Supplementary Fig. 21b) shows that the yield of the SHP CZTS nanocrystals after one single reaction is more than 3g. The obtained nanocrystals are nearly monodispersed, and most of the nanoparticles display a bullet shape morphology (Supplementary Fig. 21c-e).

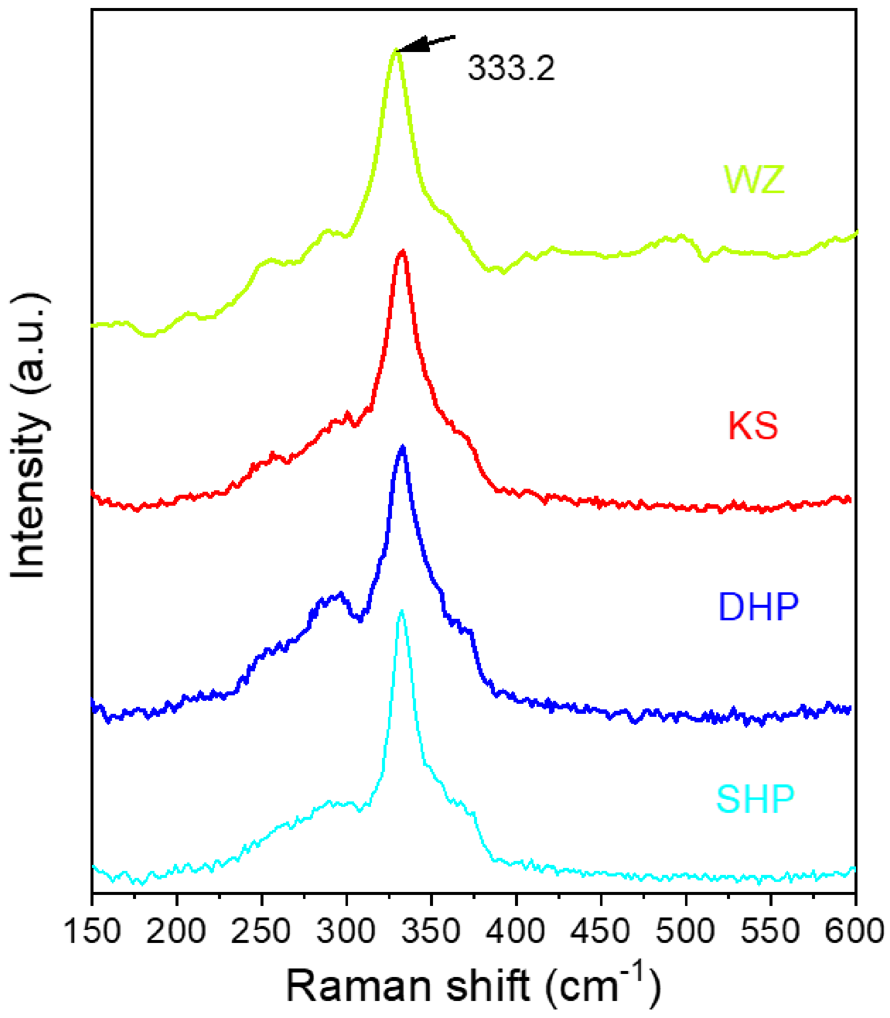

Supplementary Figure 22 | Raman spectra of the polytypic and phase-pure CZTS nanocrystals. It was used to further study the structure of the obtained polytypic and phase-pure CZTS nanocrystals because the XRD pattern cannot accurately distinguish the binary ZnS and ternary CTS. The peaks at 333.2 cm^{-1} for CZTS nanocrystals almost match the peaks of the reported CZTS[1, 2, 3]. There is no exist of the peaks for ZnS and CTS, revealing the products are CZTS nanocrystals without byproducts.

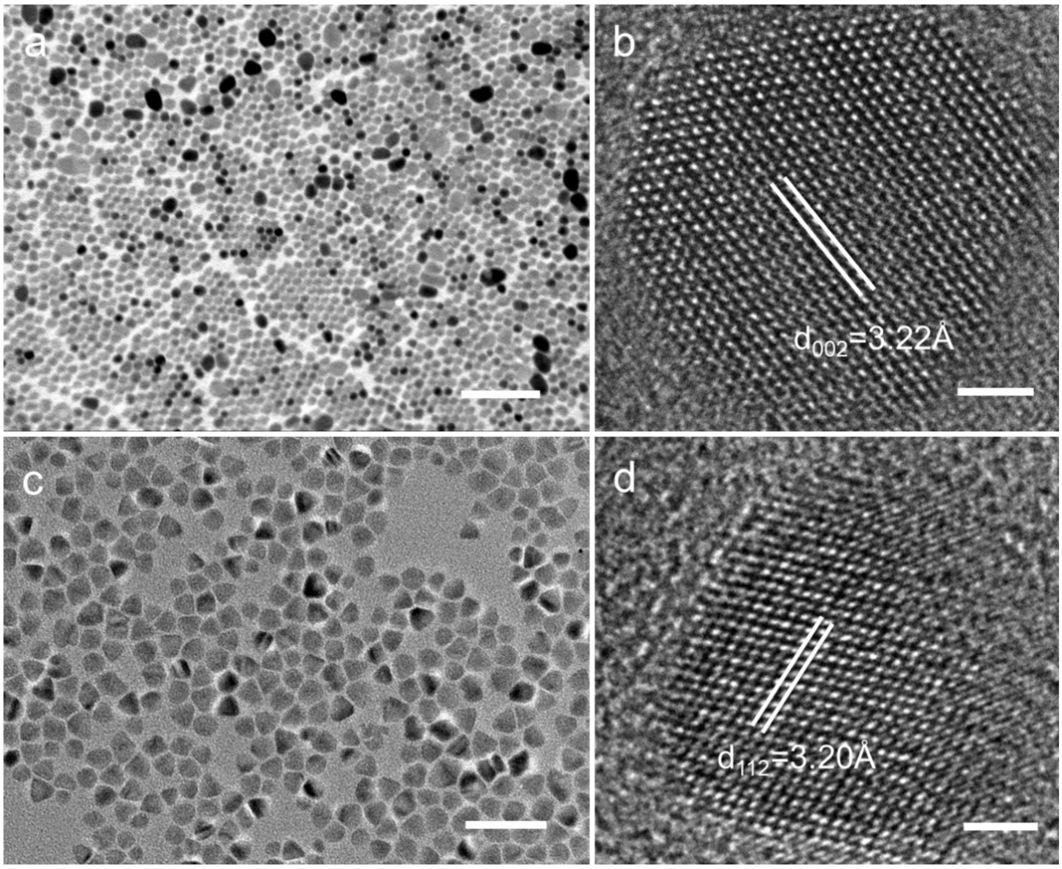

Supplementary Figure 23 | Characterization of wurztite and kesterite CZTS nanocrystalts. a-b, TEM and HTEM images of the synthesized wurtzite CZTS nanocrystals. The lattice distance of 3.22 Å in **b** can be indexed to the (002) plane of wurtzite CZTS. **c-d**, TEM and HTEM images of the synthsized kesterite CZTS nanocrystals. The lattice distance of 3.2 Å in d can be indexed to the (112) plane of kesterite CZTS. Scale bars are 100 nm for **a**, 5 nm for **b** and **d**, 50 nm for **c**, respectively.

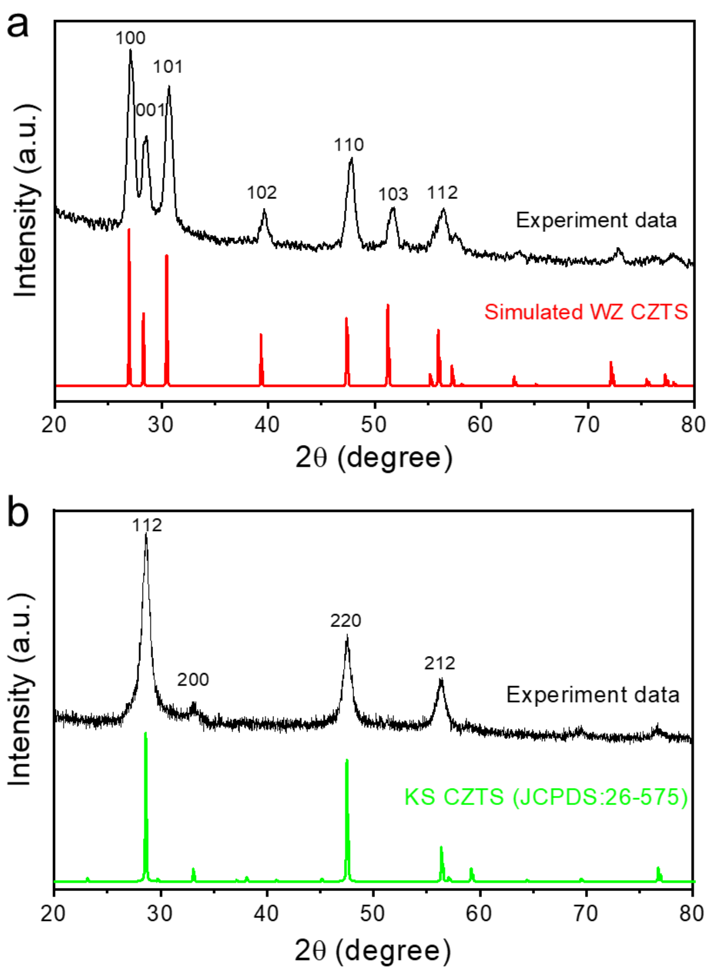

Supplementary Figure 24 | XRD pattern of the synthesized wurtzite and kesterite CZTS nanocrystals. For reference, the kesterite and simulated wurtzite XRD patterns of CZTS are shown below. The XRD pattern in **a** is perfectly matching the simulated wurtzite CZTS, certifying the synthesized CZTS nanocrystals have a pure wurtzite structure. The XRD pattern in **b** can be indexed to kesterite CZTS (JCPDS:26-575), proving the existence of pure kesterite structure.

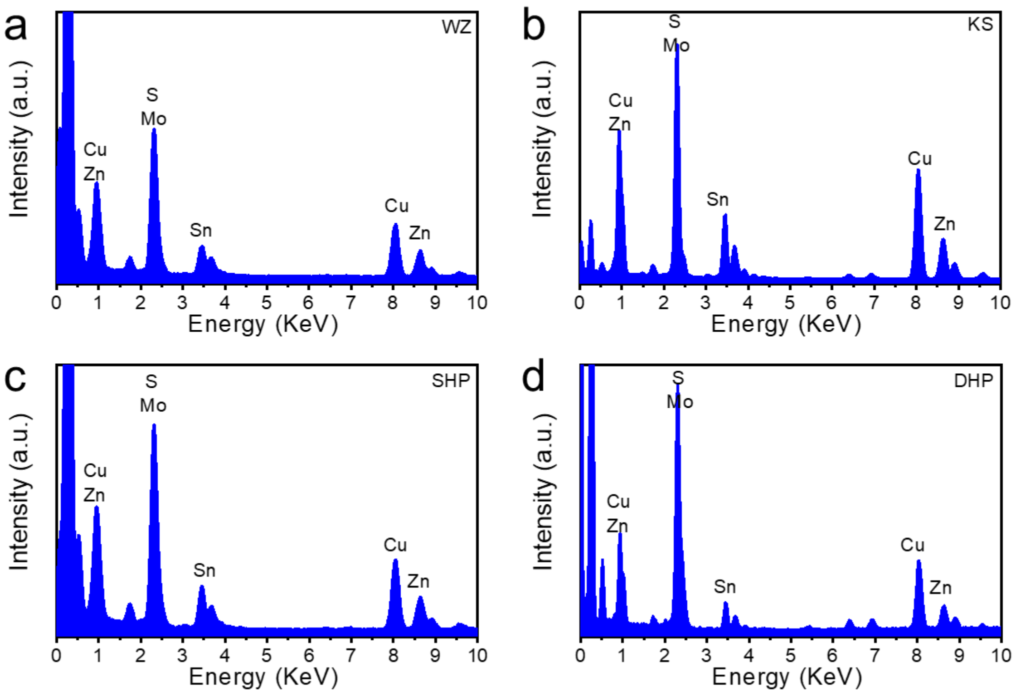

Supplementary Figure 25 | EDS spectra of the synthesized CZTS nanocrystals. **a**. Wurtzite CZTS. **b**, Kesterite CZTS. **c**, Single-homojunction polytypic CZTS. **d**, double-homojunction polytypic CZTS. The detail cation ratios are shown in Supplementary Tables 2. As a result, the synthesized polytypic (single-homojunction and double-homojunction) and phase-pure (wurtzite and kesterite) CZTS nanocrystals have the same element compositions and cation ratios.

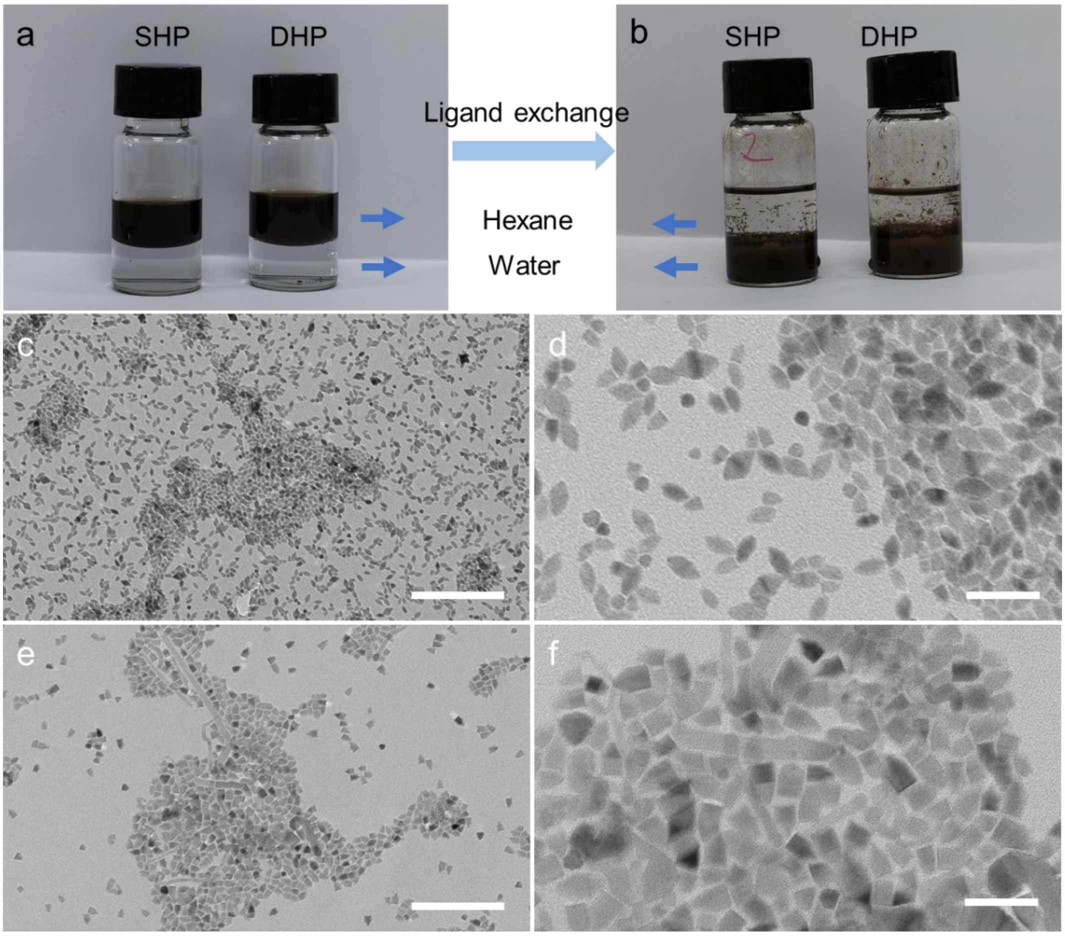

Supplementary Figure 26 | Characterization of the SHP and DHP CZTS nanocrystals after ligand exchange. **a-b**, Photographs illustrating the phase transfer of SHP and DHP CZTS nanocrystals induced by ligand exchange process. **c-d,** TEM and enlarged TEM images of DHP CZTS nanocrystals dispersed in methanol. **e-f,** TEM and enlarged TEM images of SHP CZTS nanocrystals dispersed in methanol. Scale bars are 200 nm for **c** and **e,** 50 nm for **d** and **f**, respectively.

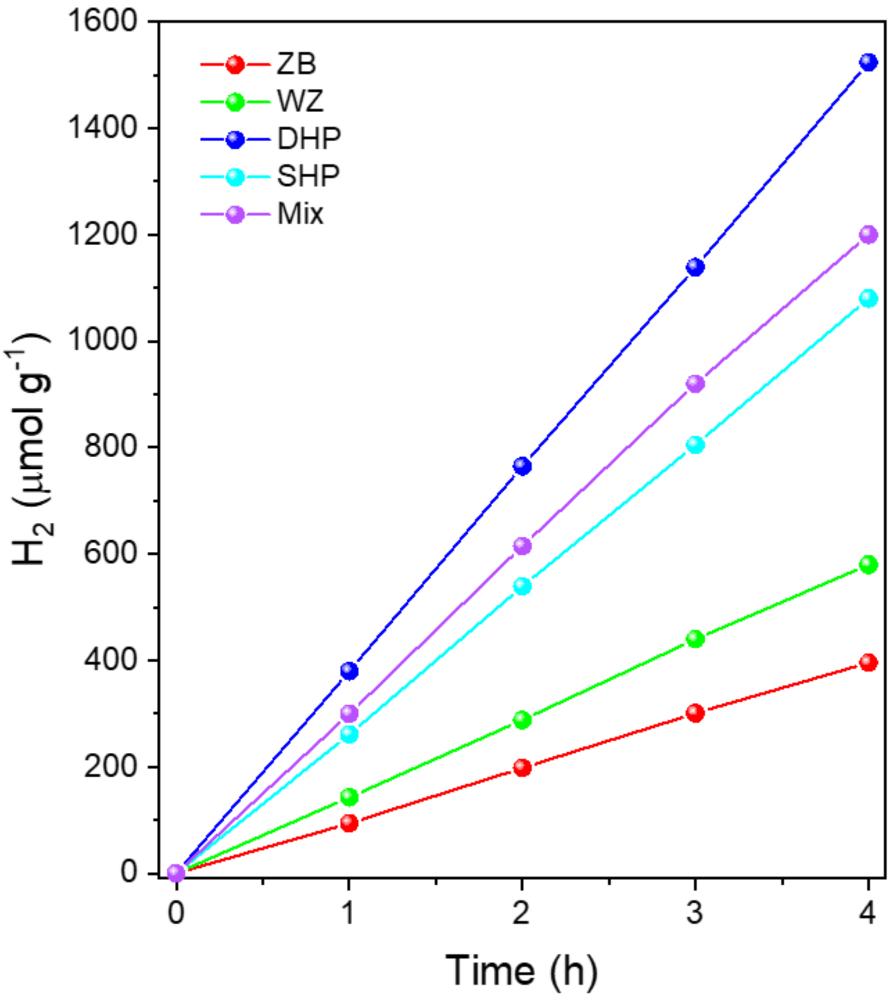

Supplementary Figure 27 | Photocatalytic H_2 activities of polytypic CZTS nanocrystals, mixed sample and phase-pure CZTS nanocrystals.

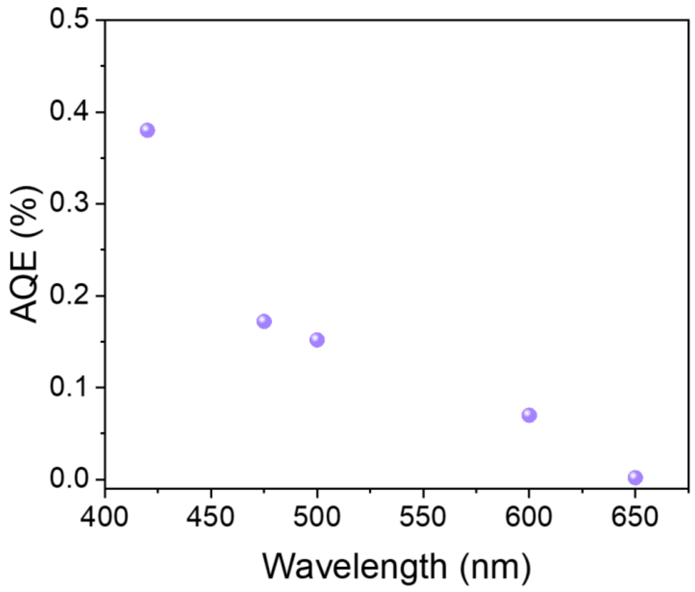

Supplementary Figure 28 | Photocatalytic efficiency of DHP CZTS nanocrystals. Apparent quantum efficiency in photocatalytic H_2 production of the DHP CZTS nanocrystals.

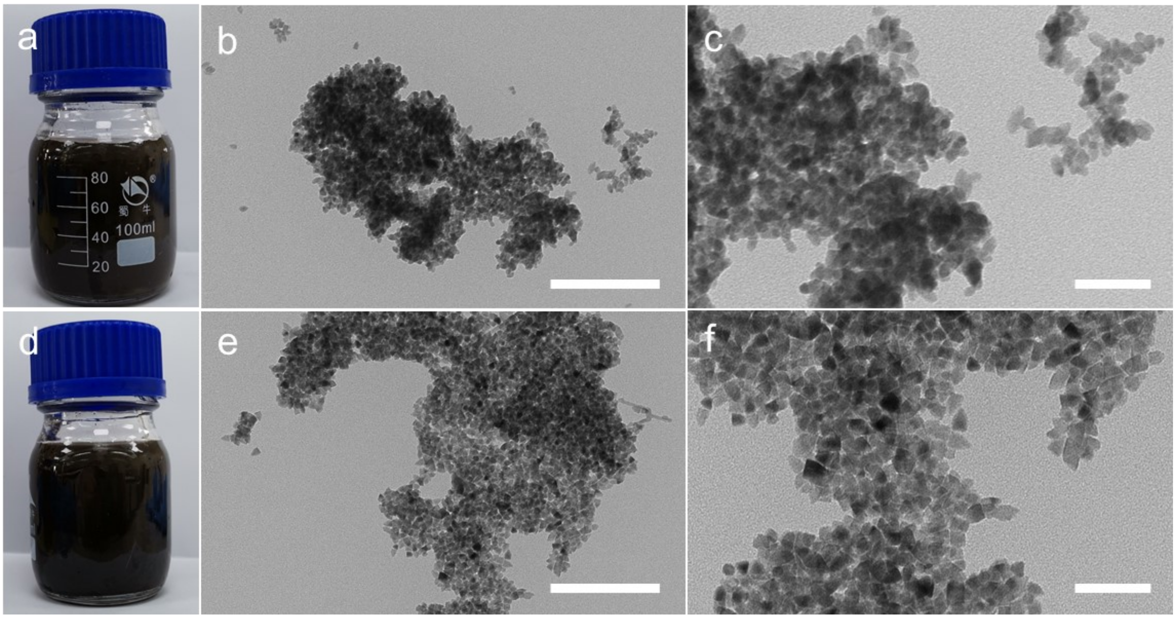

Supplementary Figure 29 | Characterization of the SHP and DHP CZTS nanocrystals after photocatalytic tests. **a**, Photograph of the DHP CZTS dispersed in water. **b-c,** TEM and enlarged TEM images of DHP CZTS nanocrystals. **d**, Photograph of the SHP CZTS dispersed in water. **e-f,** TEM and enlarged TEM images of SHP CZTS nanocrystals. Scale bars are 200 nm for **b** and **e,** 50 nm for **c** and **f**, respectively.

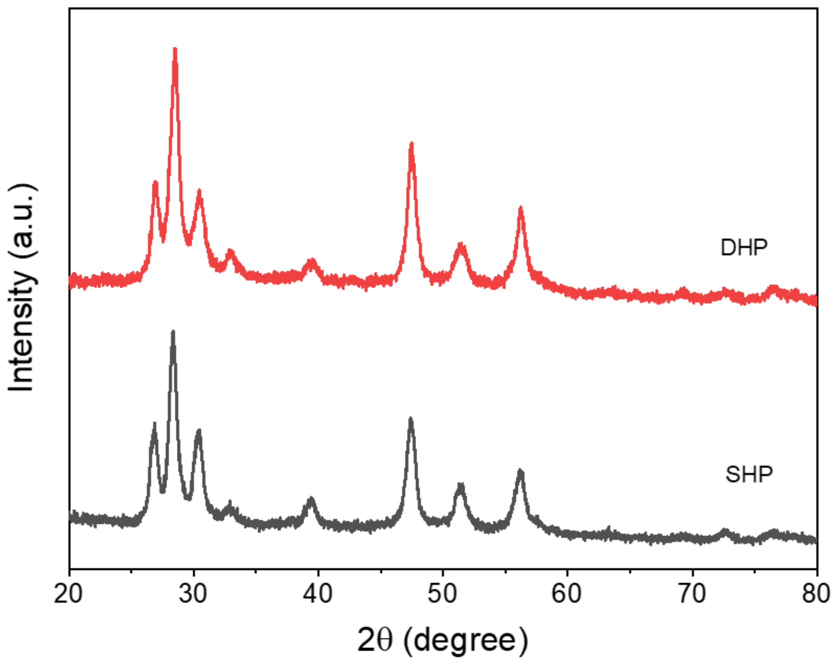

Supplementary Figure 30 | XRD patterns of the SHP and DHP CZTS nanocrystals after photocatalytic tests.

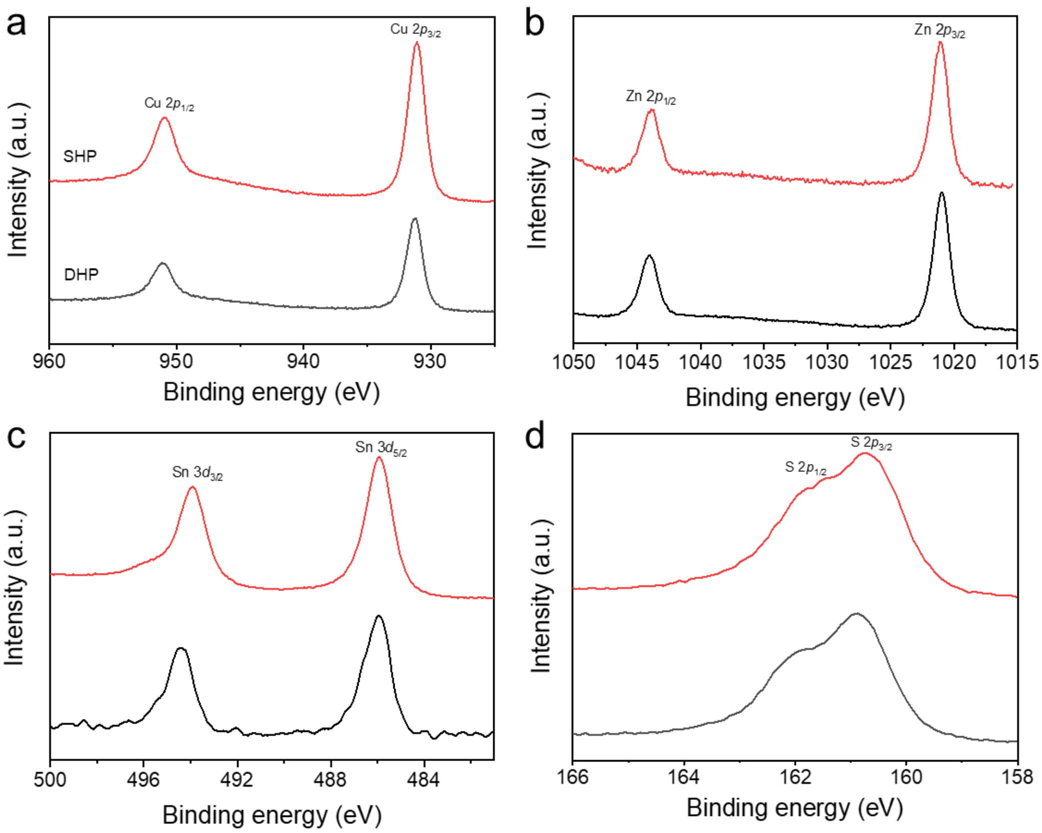

Supplementary Figure 31 | XPS spectra of the SHP and DHP CZTS nanocrystals after photocatalytic tests. **a**, Cu_{2p}. **b**, Zn_{2p}. **c**, Sn_{3d}. **d**, S_{2p}.

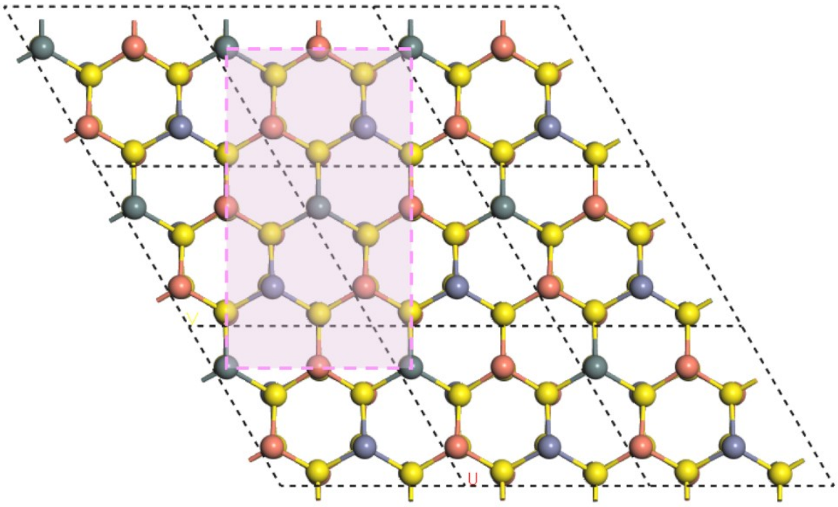

Supplementary Figure 32 | Rebuild the WZ structure along (001) facet, from hexagonal to tetragonal.

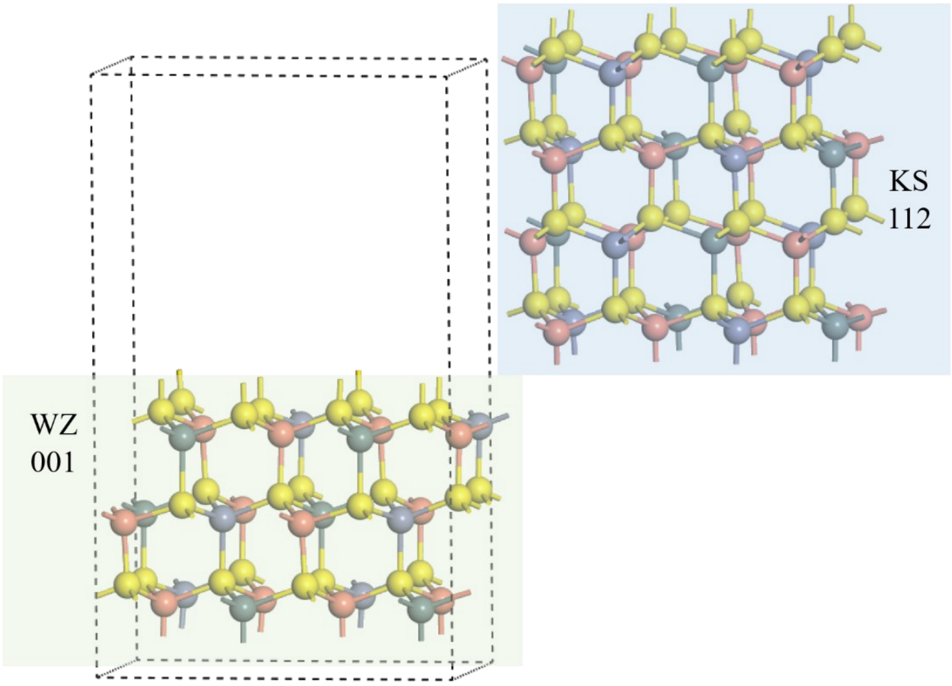

Supplementary Figure 33 | Structure models of the supercell model of KS-WZ interface

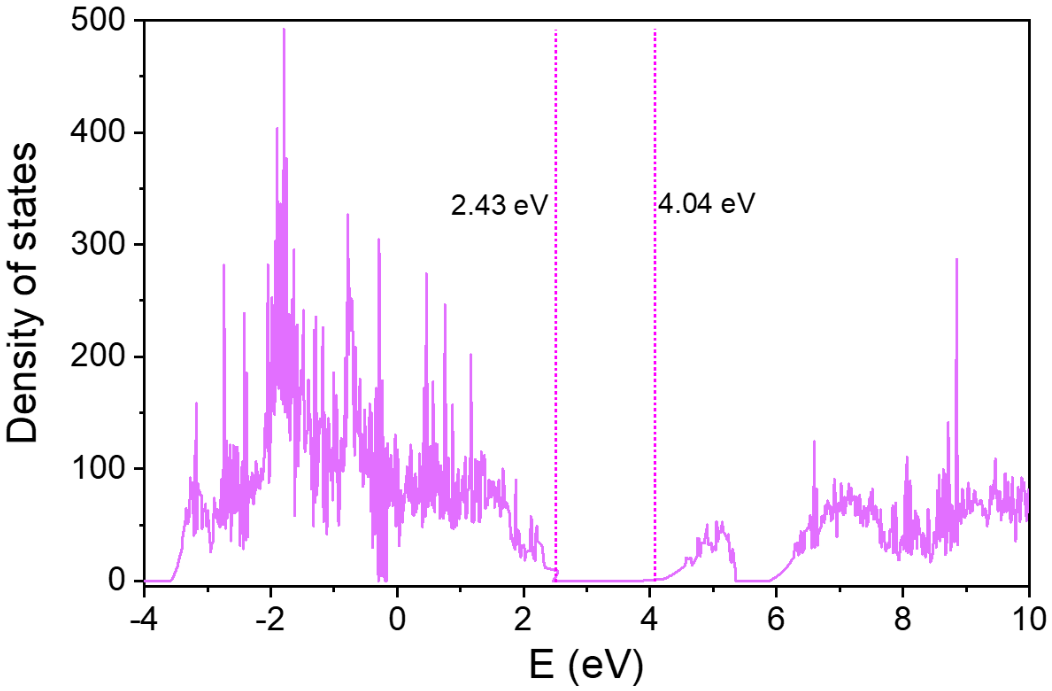

Supplementary Figure 34 | The simulated DOS of the homojunction of KS(112) and WZ(001) of CZTS.

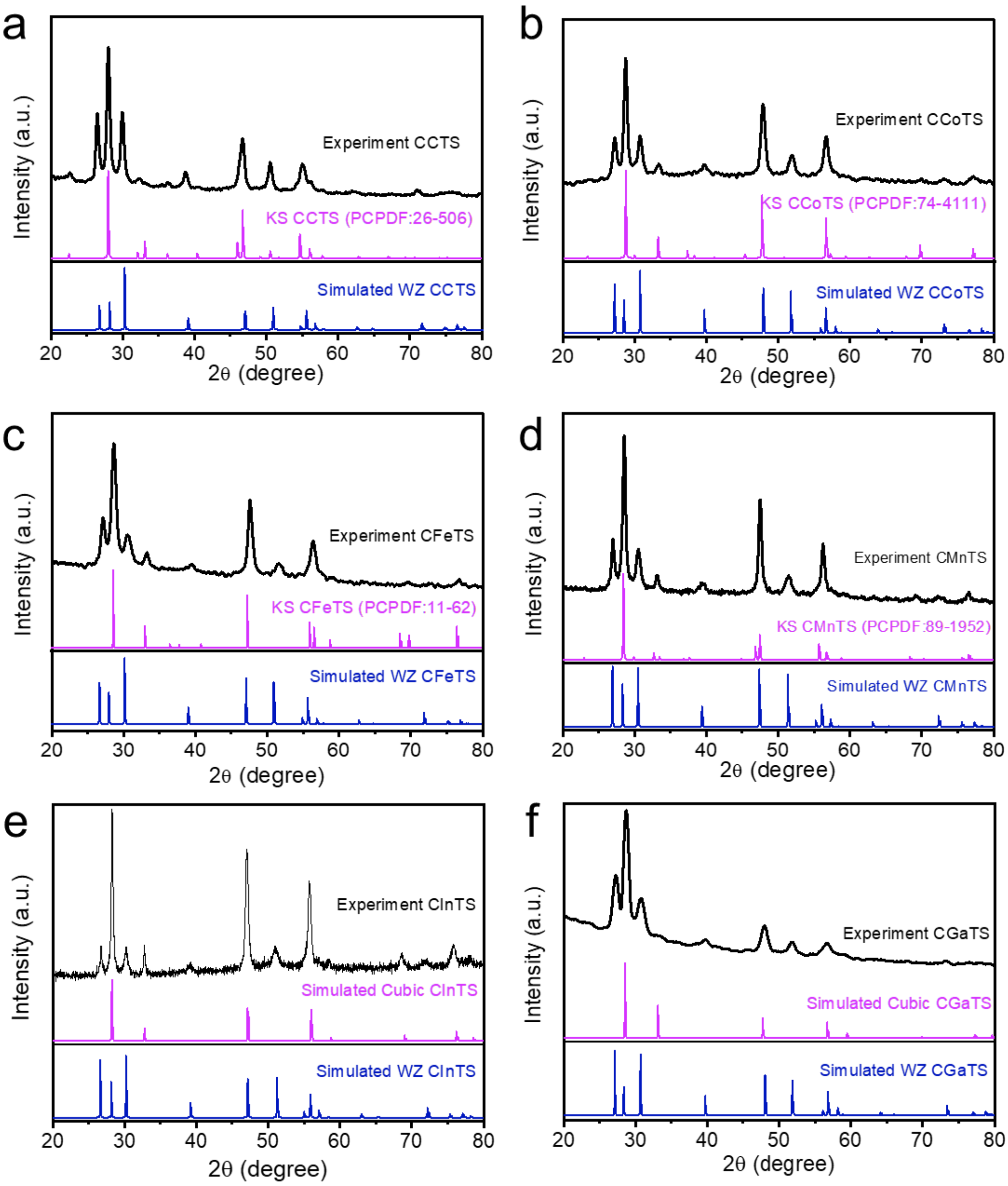

Supplementary Figure 35 | XRD patterns of the synthesized polytypic CQS nanocrystals. **a**, Polytypic CCTS. **b**, Polytypic CCoTS. **c**, Polytypic CFeTS. **d**, Polytypic CMnTS. **e**, Polytypic CInTS. **f**, Polytypic CGaTS. For reference, the kesterite and simulated wurtzite XRD patterns of different copper-base quaternary sulfides are shown below. All the XRD patterns are in the same with that of polytypic CZTS nanocrystals, which proves their polytypic structure.

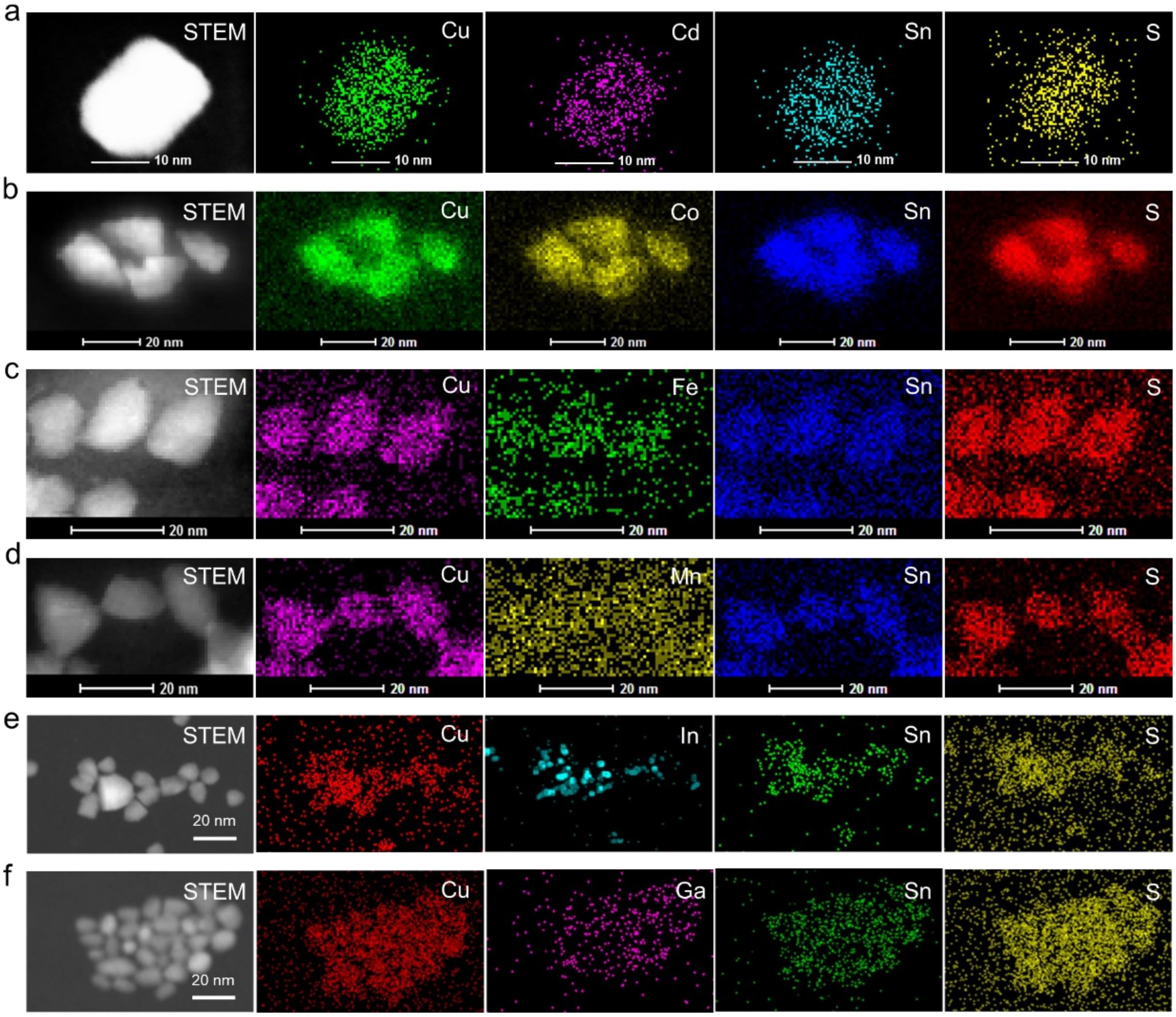

Supplementary Figure 36 | EDS element mapping of the synthesized polytypic CQS nanocrystals. **a**, Polytypic CCTS. **b**, Polytypic CCoTS. **c**, Polytypic CFeTS. **d**, Polytypic CMnTS. **e**, Polytypic CInTS. **f**, Polytypic CGaTS. EDS-mapping show that the elements homogeneously distribute in the obtained polytypic nanocrystals and the polytypic structure is not due to the element distribution.

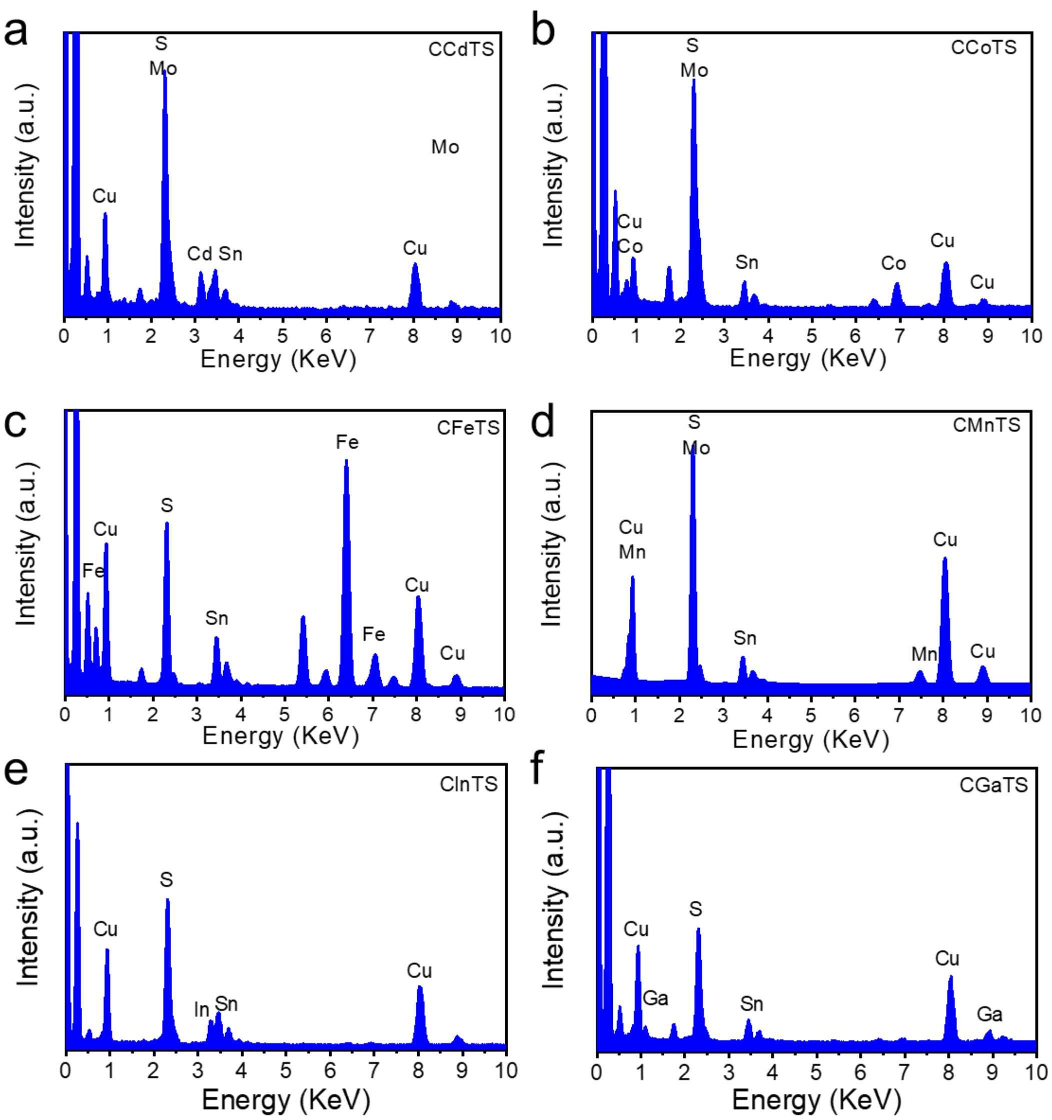

Supplementary Figure 37 | EDS spectra of the synthesized polytypic CQS nanocrystals. **a**, Polytypic CCTS. **b**, Polytypic CCoTS. **c**, Polytypic CFeTS. **d**, Polytypic CMnTS. **e**, Polytypic CInTS. **f**, Polytypic CGaTS. The detail cation ratio was listed in Supplementary Table 4.

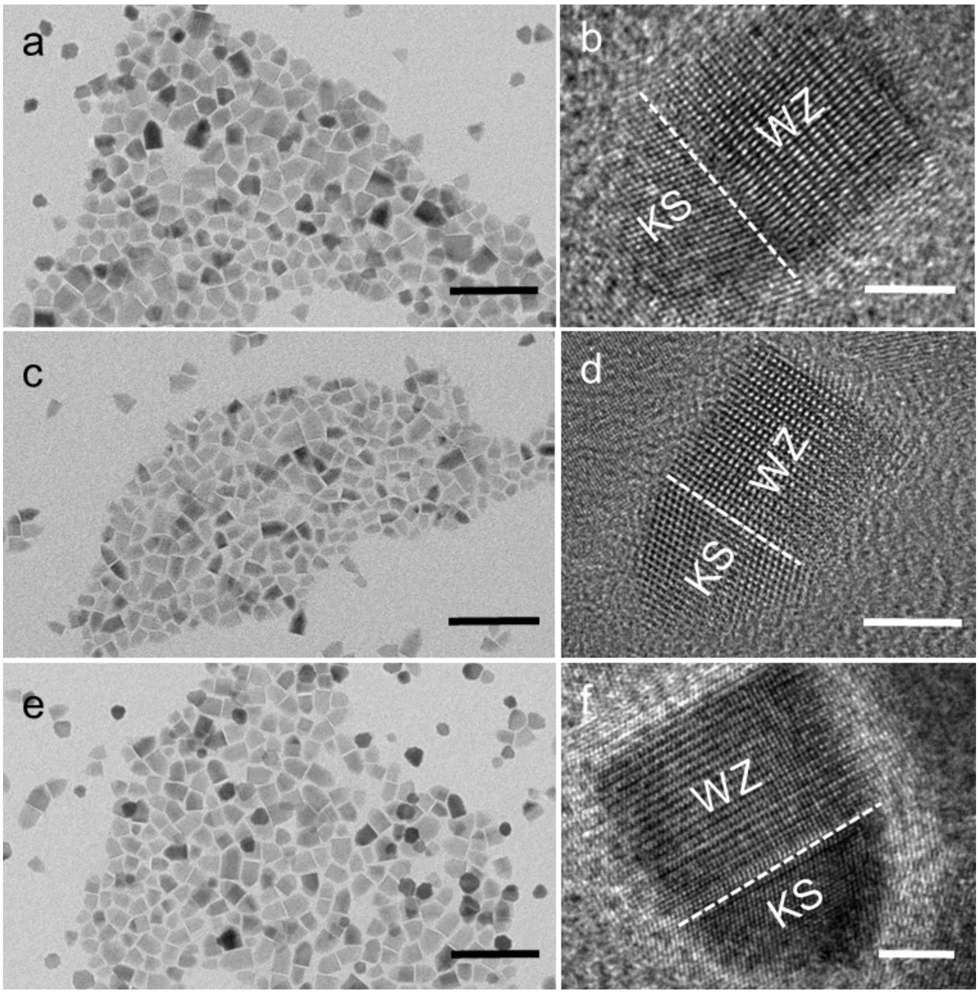

Supplementary Figure 38 | TEM and HRTEM images of the synthesized polytypic Cu-based multinary sulfide nanocrystals. **a-b**, Polytypic $Cu_2(Zn_xCd_y)SnS_4$ nanocrystals. **c-d**, Polytypic $Cu_2(Zn_xCo_y)SnS_4$ nanocrystals. **e-f**, Polytypic $Cu_2(Zn_xCd_yCo_z)SnS_4$ nanocrystals. The obtained nanocrystals possess a bullet-shaped morphology constructed with wurtzite and kesterite structure. Scale bars are 100 nm for **a**, **c** and **e**, 5 nm for **b**, **d** and **f**, respectively.

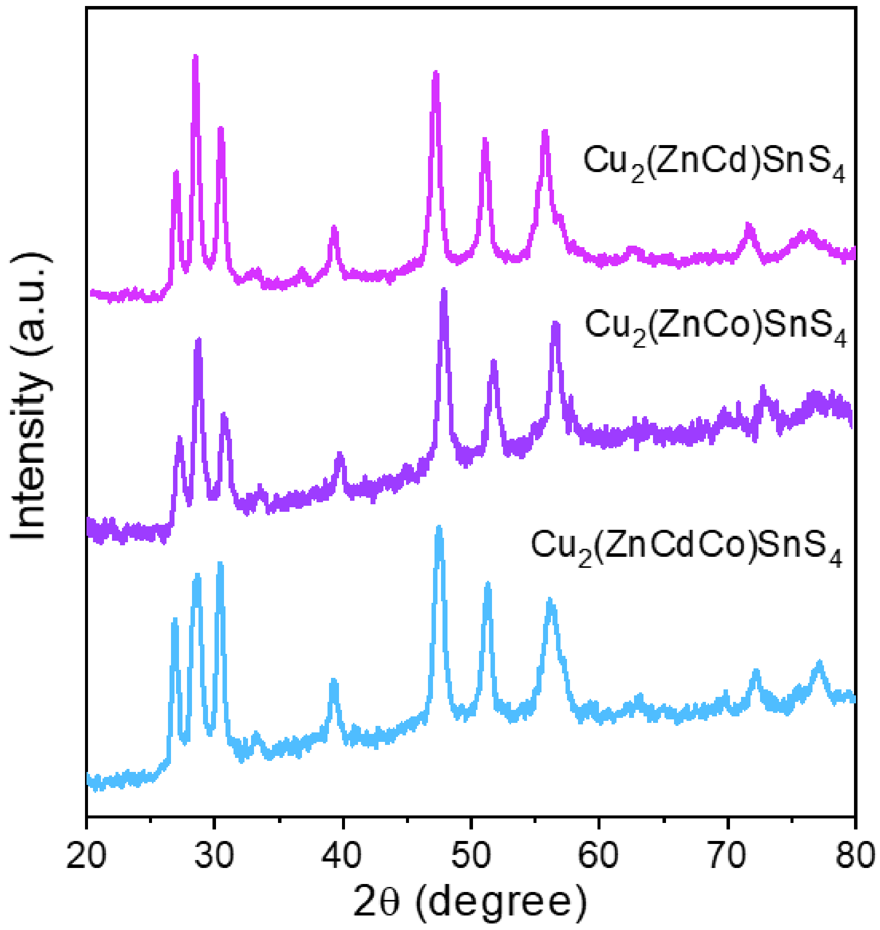

Supplementary Figure 39 | XRD patterns of the synthesized polytypic copper-based multinary sulfide nanocrystals. The XRD results also prove the presence of wurtzite and kesterite structure.

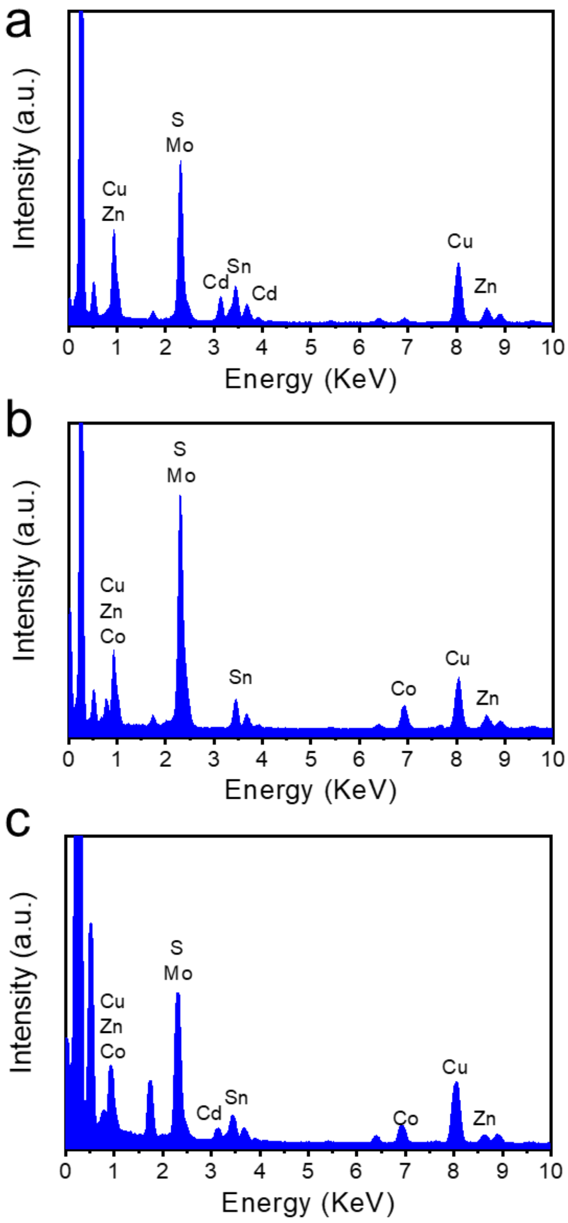

Supplementary Figure 40 | EDS spectra of the synthesized copper-based multinary polytypic sulfide nanocrystals. **a**, Polytypic $Cu_2(Zn_xCd_y)SnS_4$ nanocrystals. **b**, Polytypic $Cu_2(Zn_xCo_y)SnS_4$ nanocrystals. **c**, Polytypic $Cu_2(Zn_xCd_yCo_z)SnS_4$ nanocrystals. The detail cation ratio was listed in Supplementary Table 6.

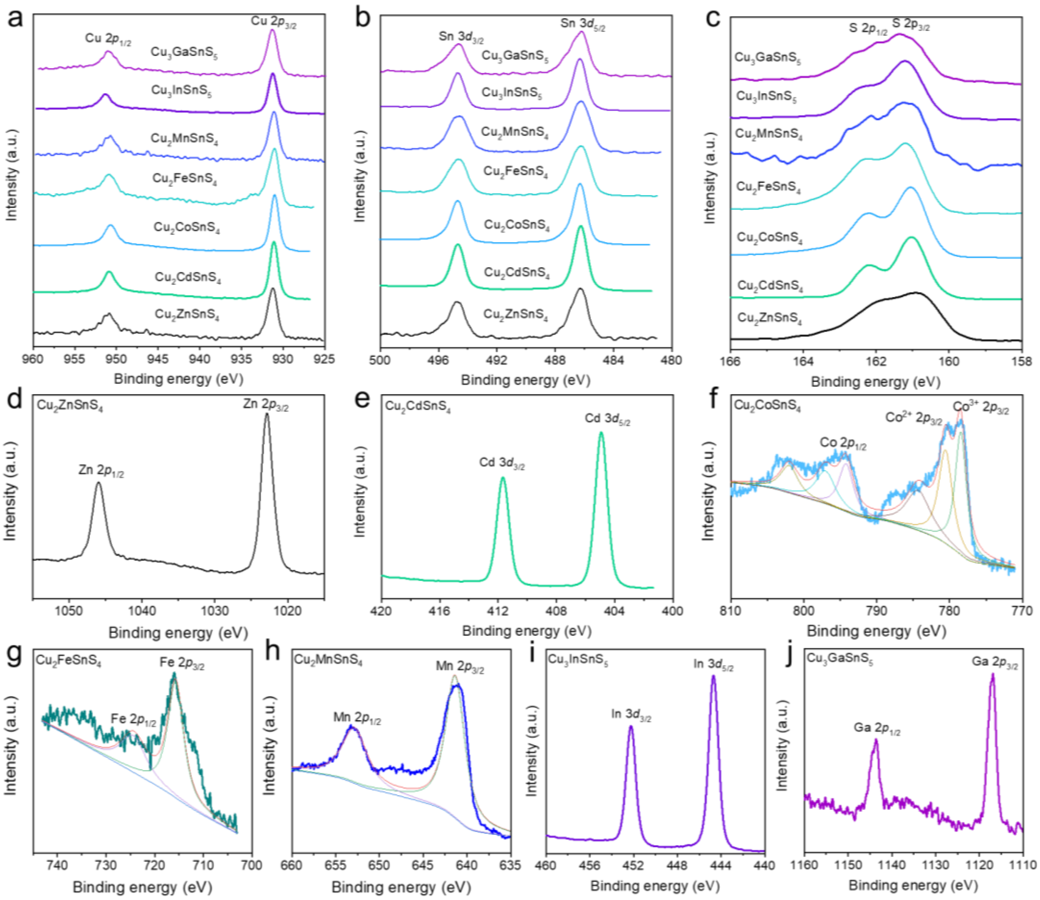

Supplementary Figure 41 | XPS spectra of the synthesized polytypic CQS nanocrystals. **a**, Cu_{2p}. **b**, Sn_{3d}. **c**, S_{2p}. **d**, Zn_{2p}, **e**,Cd_{3d}. **f**, Co_{2p}. **g**, Fe_{2p}. **h**, Mn_{2p}. **i**, In_{3d}. **j**, Ga_{2p}. The two characteristic Cu_{2p} peaks all located at 932.2 and 952 eV with a binding energy splitting of 19.8 eV, indicating the presence of the Cu(I) state (Supplementary Figure 28**a**)[4]. The Sn has the same 3d peaks located at 494.8 and 486.5 eV with a binding splitting of 8.3 eV, indicating the presence of the Sn(IV) state (Supplementary Figure 28**b**)[4]. Then, the S_{2p} peaks appeared at 161.9 and 163 eV, confirming the presence of the S(II) state (Supplementary Figure 28**c**)[4]. As the same, the XPS results (Supplementary Figure 28**d-j**) prove the existence of Zn(II), Cd(II), Co(II), Mn(II), Fe(II), In(III) and Ga(III) states in the obtianed polytypic nanocrystals, respectively[4-8]. Thus, the results of XPS analysis indicate that the phase and composition cannot influence the element oxidation state of the obtained nanocrystals.

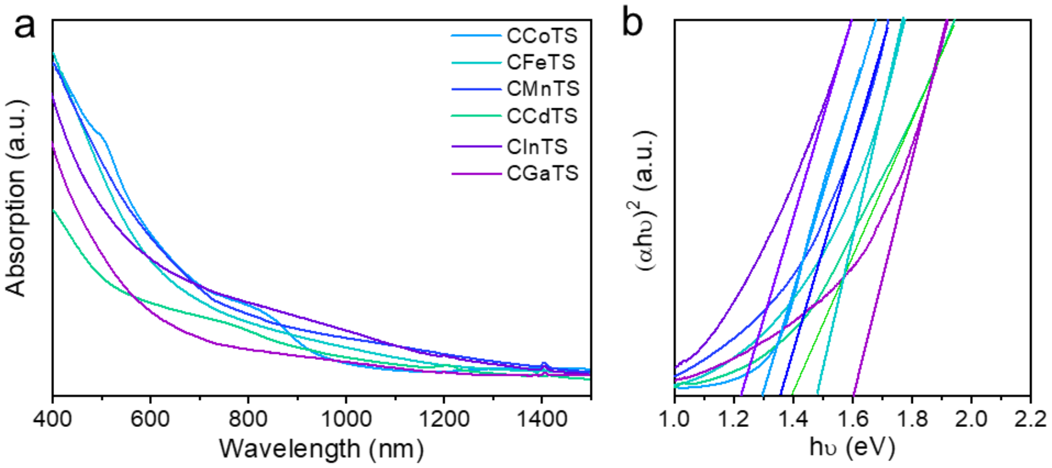

Supplementary Figure 42 | Optical properties of the synthesized polytypic CQS nanocrystals. (a) UV-Vis-IR absorption properties of the syntheiszed polytypic copper-base quaternary sulfides. (b) The graph of linear extrapolation of $(\alpha h\upsilon)^2$ versus photon energy (hυ). The detail band gap information was listed in Supplementary Table 4.

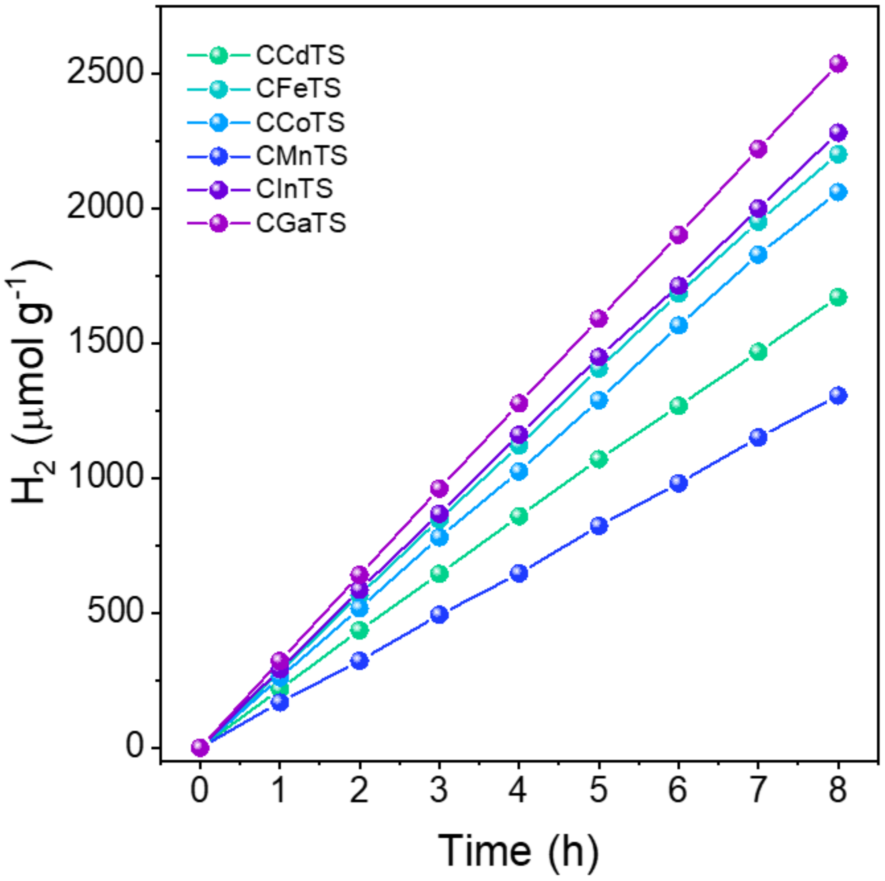

Supplementary Figure 43 | Produced of hydrogen amount after 8 h photocatalytic hydrogen evolution.

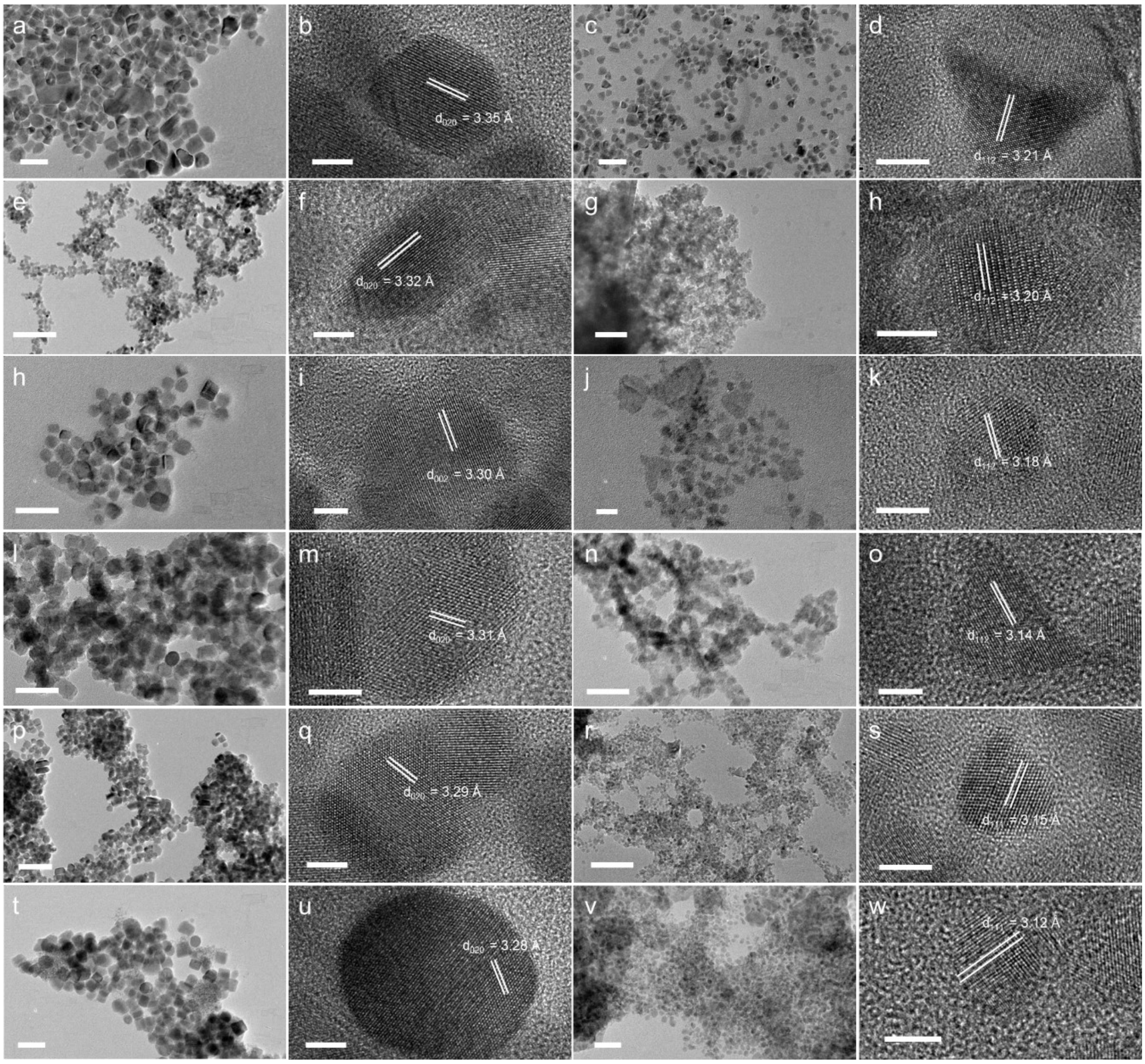

Supplementary Figure 44 | TEM and HRTEM images of the synthesized WZ and KS/WZ CQS nanocrystals. a-b, WZ CCdTS nanocrystals. **c-d,** KS CCdTS nanocrystals. **e-f,** WZ CCoTS nanocrystals. **g-h,** KS CCoTS nanocrystals. **h-I,** WZ CFeTS nanocrystals. **j-k,** KS CFeTS nanocrystals. **l-m,** WZ CMnTS nanocrystals. **n-o,** KS CMnTS nanocrystals. **p-q,** WZ CInTS nanocrystals. **r-s,** KS CInTS nanocrystals. **t-u,** WZ CGaTS nanocrystals. **v-w,** KS CGaTS nanocrystals. Scale bars are 50 nm for **a**, **c**, **h**, **l**, **n**, **t**, and **v**, 100 nm for **e**, **g**, **p**, and **r**, 20 nm for **j**, 5 nm for **b**, **d**, **f**, **h**, **i**, **k**, **m**, **o**, **q**, **s**, **u**, and **w**, respectively.

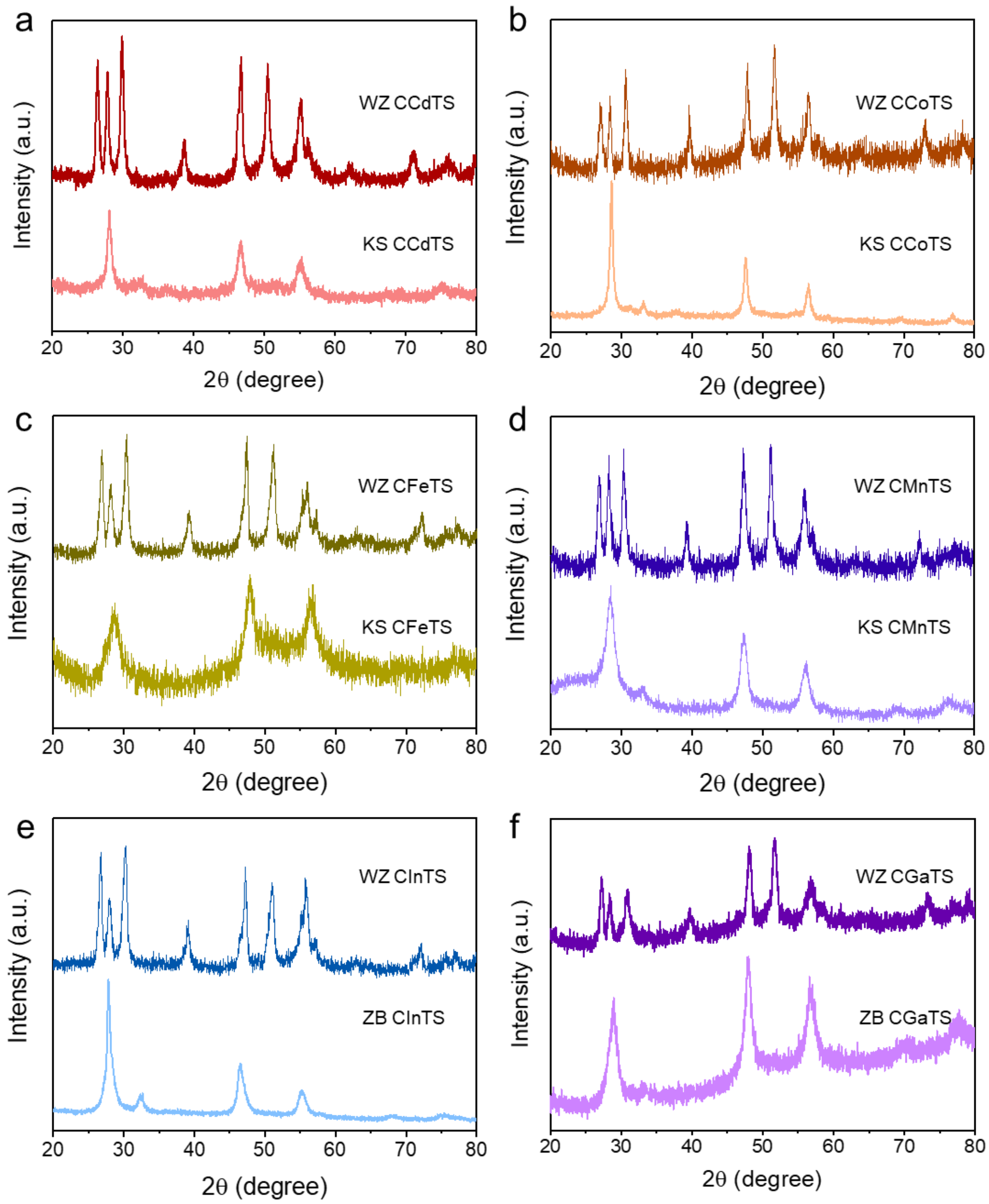

Supplementary Figure 45 | XRD patterns of the synthesized WZ and KS/WZ CQS nanocrystals. a, WZ and KS CCTS. **b**, WZ and KS CCoTS. **c**, WZ and KS CFeTS. **d**, WZ and KS CMnTS. **e**, WZ and ZB CInTS. **f**, WZ and ZB CGaTS.

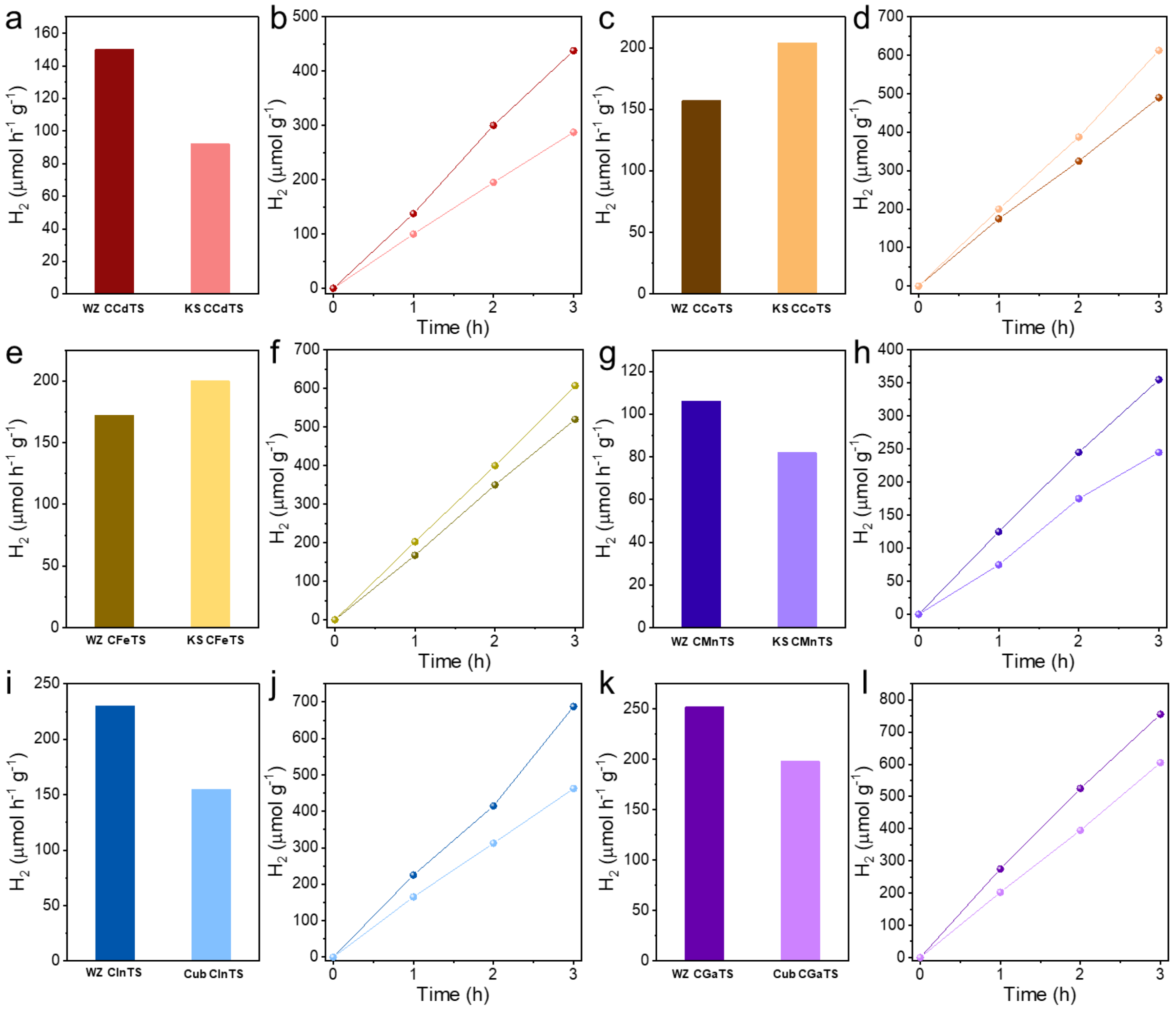

Supplementary Figure 46 | Photocatalytyic hydrogen production properties of the synthesized WZ and KS/WZ CQS nanocrystals. a-b, WZ and KS CCTS. **c-d**, WZ and KS CCoTS. **e-f**, WZ and KS CFeTS. **g-h**, WZ and KS CMnTS. **i-j**, WZ and ZB CInTS. **k-l**, WZ and ZB CGaTS.

Supplementary Table 1 | Amounts of CuCl, $Zn(AC)_2·2H_2O$, $SnCl_2·2H_2O$, 1-DDT and OLA used for synthesizing CZTS nanocrystals with different Zn content, the cation ratio and band gap of the obtained nanocrystals.

Sample	Cu (mmol)	Zn (mmol)	Sn (mmol)	OLA/1-DDT (mL)	Cu:Zn:Sn (EDS)	Band gap (eV)
**Z0**	0.28	0	0.14	10/1.5	2:0:0.99	1.38
**Z1**	0.28	0.08	0.14	10/1.5	2:0.42:0.77	1.43
**Z2**	0.28	0.14	0.14	10/1.5	2:0.68:0.72	1.46
**Z4**	0.28	0.28	0.14	10/1.5	2:0.97:0.97	1.55
**Z5**	0.28	0.35	0.14	10/1.5	2:1.37:0.97	1.59
**Z6**	0.28	0.42	0.14	10/1.5	2:1.79:0.98	1.63

Supplementary Table 2 | Amounts of precursors used for synthesizing CZTS nanocrystals, the cation ratio and band gap of the obtained nanocrystals.

Sample	Cu (mmol)	Zn (mmol)	Sn (mmol)	Cu:Zn:Sn (EDS)	Band gap (eV)
**WZ**	1.8	1.3	0.7	2:0.79:0.99	1.55
**KS**	0.28	0.21	0.14	2:0.77:1.02	1.44
**SHP**	0.28	0.21	0.14	2:0.82:0.95	1.47
**DHP**	0.28	0.21	0.14	2:0.80:0.97	1.51

Supplementary Table 3 | Comparison of the photocatalytic H_2 production performances for the representative CZTS-based photocatalysts.

Photocatalyst	Weight (mg)	Hole scavenger	H_2 evolution rate (μmol h^{-1} g^{-1})	Ref.
WZ- NPs	10 mg	0.1 M Na_2S and 0.1 M $NaSO_3$	130	9
WZ-nanorods	10 mg	0.35 M Na_2S and 0.25 M Na_2SO_3	44	10
WZ-nanoplates	10 mg	0.35 M Na_2S and 0.25 M Na_2SO_3	14	10
WZ- NPs	10 mg	0.4 M Na_2S and 0.3 M Na_2SO_3	64.5	11
KS- NPs	40 mg	0.4 M Na_2S and 0.3 M Na_2SO_3	82.3	12
KS- NPs	5 mg	0.35 M Na_2S and 0.25 M Na_2SO_3	25	13
WZ-NPs	40mg	0.4 M Na_2S and 0.3 M Na_2SO_3	21.2	14
KS-NPs	40mg	0.4 M Na_2S and 0.3 M Na_2SO_3	54.8	14
KS-NPs	20mg	0.4 M Na_2S and 0.3 M Na_2SO_3	40.6	15
KS-NPs	40 mg	0.4 M Na_2S and 0.3 M Na_2SO_3	70	16
KS-NPs	20 mg	0.1 M Na_2S and 0.1 M Na_2SO_3	60	17
KS- NPs	30 mg	0.35 M Na_2S and 0.25 M Na_2SO_3	98	This work
WZ- NPs	30 mg	0.35 M Na_2S and 0.25 M Na_2SO_3	145	This work
SHP	30 mg	0.35 M Na_2S and 0.25 M Na_2SO_3	270	This work
DHP	30 mg	0.35 M Na_2S and 0.25 M Na_2SO_3	381	This work
CZTS-Au	10 mg	0.1 M Na_2S and 0.1 M $NaSO_3$	805	9
CZTS-Pt	10 mg	0.1 M Na_2S and 0.1 M $NaSO_3$	1020	9
Au@CZTS	10 mg	0.35 M Na_2S and 0.25 M Na_2SO_3	102	10
CZTS-CdS	10 mg	0.4 M Na_2S and 0.3 M Na_2SO_3	937.6	11
CZTS-SiO_2	20 mg	0.4 M Na_2S and 0.3 M Na_2SO_3	102.6	15
CZTS-CdS	40 mg	0.4 M Na_2S and 0.3 M Na_2SO_3	4600	16
CZTS-PtCo	10mg	0.1 M Na_2S and 0.1 M $NaSO_3$	1850	18
CZTS-MoS_2	20 mg	0.1 M Na_2S and 0.1 M Na_2SO_3	470	17
CZTS-MoS_2-GO	5 mg	0.35 M Na_2S and 0.25 M Na_2SO_3	108	13

Supplementary Table 4 | Lattice constants matching between the KS (112) and WZ (001) facets.

Lattice constant	a (nm)	b (nm)
**KS (112)**	0.761	1.317
**WZ (001)**	0.762	1.328

Supplementary Table 5 | Amounts of precursors, 1-DDT and OLA used for synthesizing the polytypic CQS nanocrystals, the cation ratio and band gap of the obtained nanocrystals.

Sample	M (mmol)	Cu/Sn (mmol)	OLA/DDT (mL)	(EDS)	Band gap (eV)
**CCdTS**	$Cd(AC)_2$ (0.14)	0.28/014	10/1.5	$Cu_2Cd_{0.69}Sn_{0.94}S_3$	1.39
**CCoTS**	$Co(AC)_2$ (0.14)	0.28/0.14	10/1.5	$Cu_2Co_{0.91}Sn_{1.05}S_{3.91}$	1.31
**CFeTS**	$Fe(AC)_2$ (0.12)	0.28/0.14	10/1.5	$Cu_2Fe_{0.44}Sn_{0.91}S_{3.12}$	1.48
**CMnTS**	$Mn(AC)_2$ (0.12)	0.28/0.14	10/1.5	$Cu_2Mn_{0.48}Sn_{0.77}S_{3.02}$	1.35
**CInTS**	$In(acac)_3$ (0.14)	0.28/0.14	10/1.5	$Cu_3In_{1.1}Sn_{1.2}S_{5.6}$	1.25
**CGaTS**	$Ga(acac)_3$ (0.14)	0.28/0.14	10/1.5	$Cu_3Ga_{0.48}Sn_{1.1}S_{4.5}$	1.60

Supplementary Table 6 | Space group, lattice parameter and atom positions of simulated wurtzite and cubic CQS semiconductors.

Material	Space group	Lattice parameter		S			Cu/M/Sn		
		a=b/Å	c/Å	x	y	z	x	y	z
CZTS	P63mc	3.8387	6.3388	0.333	0.667	0	0.333	0.667	0.375
CTS	P63mc	3.8253	6.3258	0.333	0.667	0	0.333	0.667	0.375
CCdTS	P63mc	3.9257	6.4458	0.333	0.667	0	0.333	0.667	0.375
CCoTS	P63mc	3.8420	6.3458	0.333	0.667	0	0.333	0.667	0.375
CFeTS	P63mc	3.8560	6.3641	0.333	0.667	0	0.333	0.667	0.375
CMnTS	P63mc	3.8178	6.3163	0.333	0.667	0	0.333	0.667	0.375
CInTS	P63mc	3.8720	6.3544	0.333	0.667	0	0.333	0.667	0.375
CGaTS	P63mc	3.810	6.317	0.333	0.667	0	0.333	0.667	0.375
CInTS	F-43m	5.50	5.50	0.25	0.25	0.25	0	0	0
CGaTS	F-43m	5.47	5.47	0.25	0.25	0.25	0	0	0

Supplementary Table 7 | Amounts of precursors, 1-DDT and OLA used for synthesizing the polytypic copper-based multinary nanocrystals, the cation ratio and band gap of the obtained nanocrystals.

Sample	Cu/Sn	Cd	Co (mmol)	Mn	OLA/DDT (mL)	EDS
**CZCdTS**	0.28/0.14	0.14	0	0.14	10/1.5	$Cu_2Zn_{0.5}Cd_{0.7}SnS_{4.1}$
**CZCoTS**	0.28/0.14	0	0.14	0.14	10/1.5	$Cu_2Zn_{0.5}Co_{0.8}SnS_{4.4}$
**CZCdCoTS**	0.28/0.14	0.1	0.1	0.1	10/1.5	$Cu_2Zn_{0.3}Cd_{0.4}Co_{0.5}Sn_{0.8}S_{3.7}$

Supplementary Table 8 | Amounts of precursors, 1-DDT and OLA used for synthesizing the WZ CQS nanocrystals

Sample	M (mmol)	$Cu(acac)_2$/$Sn(acac)_2Cl_2$ (mmol)	OLA (mL)	1-DDT (mL)	t-DDT (mL)	Reaction Temperature
**WZ CCdTS**	$Cd(acac)_2$ (0.5)	1/0.5	12	0.37	2.63	220 ºC
**WZ CCoTS**	$Co(acac)_3$ (0.5)	1/0.5	12	0.37	2.63	210 ºC
**WZ CFeTS**	$Fe(acac)_3$ (0.4)	1/0.5	12	0.37	2.63	210 ºC
**WZ CMnTS**	$Mn(acac)_3$ (0.4)	1/0.5	12	0.37	2.63	210 ºC
**WZ CInTS**	$In(acac)_3$ (0.5)	1.5/0.5	12	0.25	2.75	220 ºC
**WZ CGaTS**	$Ga(acac)_3$ (0.5)	1.5/0.5	12	0.25	2.75	220 ºC

Supplementary Table 9 | Amounts of precursors, and OLA used for synthesizing the KS/ZB CQS nanocrystals

Sample	M (mmol)	$CuCl/SnCl_2$ (mmol)	OLA (mL)	S (mmol)
**KS CCdTS**	$Cd(AC)_2$ (0.28)	0.56/0.28	15	3
**KS CCoTS**	$Co(AC)_2$ (0.28)	0.56/0.28	15	3
**KS CFeTS**	$Fe(AC)_2$ (0.24)	0.56/0.28	15	3
**KS CMnTS**	$Mn(AC)_2$ (0.24)	0.56/0.28	15	3
**KS CInTS**	$In(AC)_3$ (0.2)	0.6/0.2	15	3
**KS CGaTS**	$Ga(acac)_3$ (0.2)	0.6/0.2	15	3

Supplementary Methods

Chemicals

Ethanol (99.7%), Methanol (99.7%), hexane (97%), 1-dodecanethiol (97%, 1-DDT), tert-dodecylmercaptan (98%, t-DDT), CuCl (97%), $CuCl_2 \cdot 2H_2O$ (99.0%), $Zn(CH_3COO)_2 \cdot 2H_2O$ ($Zn(AC)_2 \cdot 2H_2O$ 99.0%), ZnO (99.0%), $Cd(CH_3COO)_2 \cdot 2H_2O$ ($Cd(AC)_2 \cdot 2H_2O$, 98.5%), $Co(CH_3COO)_2 \cdot 4H_2O$ ($Co(AC)_2 \cdot 4H_2O$, 99.0%), $SnCl_2 \cdot 2H_2O$ (98.0%), $SnCl_4 \cdot 5H_2O$ (99.0%), Na_2SO_3 (97%), KOH (85.0%), chloroform (99.0%), dodecylamine (DDA, 97%), methanol (99.7%), tetrachloroethylene (97.0%), tetrahydrofuran (THF, 99.8%) were purchased from Sinopharm Chemical Reagent Co. Ltd (Shanghai). $Mn(CH_3COO)_2$ ($Mn(AC)_2$, 98.0%), $Fe(CH_3COO)_2$ ($Fe(AC)_2$, 95.0%), hexadecanethiol (HDT, 97%) and 3-mercaptopropionic acid (MPA, 99%) were purchased from J&K. Indium(III) 2,4-pentanedionate ($In(acac)_3$, 98%), Iron(III) acetylacetonate ($Fe(acac)_3$, 99%), Cadmium acetylacetonate ($Cd(acac)_2$, 99.9%), Cobalt(III) acetylacetonate ($Co(acac)_3$, 99%), Manganese(III) acetylacetonate ($Mn(acac)_3$, 99%), and Gallium(III) 2,4-pentanedionate ($Ga(acac)_3$, 99.9%) were purchased from Alfa Aesar. Oleylamine (OLA, 80%-90%), 1-octadecene (ODE, 80%-90%), Octadecylamine (ODA, 90%), 1-hexadecylamine (HDA, 90%), 1-hexanethiol (HNT, 96%), Copper acetylacetonate ($Cu(acac)_2$, 97%), Tin(IV) bis(acetylacetonate) dichloride ($Sn(acac)_2Cl_2$, 98%) and $Na_2S \cdot 2H_2O$ (98%) was purchased from Aladdin Reagent Co. Ltd (Shanghai). All chemical reagents were used as received without further purification.

Synthesis of wurtzite CZTS nanocrystals. Using a modification of a published procedure[46], $CuCl_2 \cdot 2H_2O$ (1.8 mmol), ZnO (1.3 mmol), and of $SnCl_4 \cdot 5H_2O$ (0.7 mmol) were dissolved in 1 mL of THF. Afterward, 3 mL of OLA and 10 mL of ODE were added to the reaction mixture.

The solution was heated under nitrogen flow to 175 °C and maintained at this temperature for 1.5 h to ensure the removal of traces of low-boiling point impurities and water. After purging, the mixture was cooled to 100 °C and 4 mL of t-DDT and 0.4 mL of 1-DDT were added through a syringe. The solution then was heated to 250 °C during 10 min and maintained at this temperature for 1 h. Wurtzite CZTS NCs were thoroughly purified by multiple precipitation and redispersion steps, using 2-propanol and chloroform. Finally, CZTS NCs were dissolved in THF and the solution was centrifuged at 5550 g for 8 min to precipitate poorly soluble unreacted metal complexes and large Zn-rich particles.

Synthesis of the other WZ CQS nanocrystals. For the synthesis of WZ CCdTS nanocrystals, 1 mmol of $Cu(acac)_2$, 0.5 mmol of $Sn(acac)_2Cl_2$, 0.5 mmol of $Cd(acac)_2$, and 10 mL of OLA were degassed at room temperature for 15 min. The mixture was subsequently heated to 160 °C under N_2 atmosphere, and a mixture of 0.37 mL of 1-DDT and 2.23 mL of t-DDT was quickly injected into the solution. WZ CCdTS nanocrystals were thoroughly purified by multiple precipitation and redispersion steps, using 2-propanol and hexane. The reaction mixture was then heated up to 225 °C under an N_2 atmosphere in 30 min. WZ CCdTS, CCoTS, CFeTS, CInTS, and CGaTS are synthesized through the same procedure for WZ CCdTS nanocrystals. The detail amounts of the precursors were listed in Supplementary Table 5.

Synthesis of kesterite CZTS nanocrystal. In a typical synthesis, CuCl (0.28 mmol), $Zn(CH_3COO)_2{\cdot}2H_2O$ (0.21 mmol) and $SnCl_2{\cdot}2H_2O$ (0.14mmol) were dissolved in 10 mL of OLA in a 25 mL three-neck flask in air and then heated up to 180 °C at a heating rate of 10 °C/min. S-OLA (1.0 mmol dissolved in 5 mL of OLA) was added through a syringe. The solution then was heated to 280 °C during 10 min and maintained at this temperature for 1 h.

Then, the flask was removed from the heating mantle and naturally cooled down. The black product was collected and centrifuged at 3552 g for 5 min and the upper clear solution was discarded.

Synthesis of other KS/ZB CQS nanocrystals. The synthesis method is the same as that for KS CZTS nanocrystals with the replacement of $Zn(AC)_2·2H_2O$ with $Cd(AC)_2·2H_2O$, $Co(AC)_2·4H_2O$, $Mn(AC)_2$, $Fe(AC)_2$, $In(AC)_3$ and $Ga(acac)_3$. The detail amounts of the precursors were listed in Supplementary Table 6.

Large-scale synthesis of SHP CZTS nanocrystals. In a large scale synthesis of SHP CZTS nanocrystals, CuCl (22.4 mmol), $Zn(AC)_2·2H_2O$ (16.8 mmol) and $SnCl_2·2H_2O$ (11.2 mmol) were dissolved in a mix solution with 100 mL of OLA and 20 mL of 1-DDT in a 250 mL three-neck flask in air and vacuumed at 120 ºC for 60 min. Then, the mix solution was heated up to 280 ºC at a heating rate of 10 ºC/min and kept at 280 ºC for 60 min. Thereafter, the flask was removed from the heating mantle and naturally cooled down. The black product was collected and centrifuged at 3552 g for 5 min and the upper clear solution was discarded.

The apparent quantum efficiency (AQE) test. AQE was measured under the identical photocatalytic reactions by using 420 nm, 475 nm, 500 nm, 600 and 650 nm band pass filters. The light intensity was calibrated using an irradiation meter. The AQE was calculated by equation (1):

$$AQE = 2N_{H}/N_{P}*100\% \quad (1)$$

where N_H denotes the number of H_2 molecules and N_P denotes the number of incident photons.

Supplementary References

1. Tan, J. M., Lee, Y. H., Pedireddy, S., Baikie, T., Ling, X. Y. & Wong, L. H. Understanding the synthetic pathway of a single-phase quarternary semiconductor using surface-enhanced Raman scattering: a case of wurtzite Cu_2ZnSnS_4 nanoparticles. *J. Am. Chem. Soc.* **136**, 6684-6692 (2014).
2. Shavel, A., Cadavid, D., Ibanez, M., Carrete, A. & Cabot, A. Continuous production of Cu_2ZnSnS_4 nanocrystals in a flow reactor. *J. Am. Chem. Soc.* **134**, 1438-1441 (2012).
3. Mainz, R. *et al.* Phase-transition-driven growth of compound semiconductor crystals from ordered metastable nanorods. *Nat. Commun.* **5**, 3133 (2014).
4. Singh, A., Geaney, H., Laffir, F. & Ryan, K. M. Colloidal Synthesis of Wurtzite Cu_2ZnSnS_4 Nanorods and Their Perpendicular Assembly. *J. Am. Chem. Soc.* **134**, 2910-2913 (2012).
5. Cui, Y., Wang, G. & Pan, D. Synthesis and photoresponse of novel Cu_2CdSnS_4 semiconductor nanorods. *J. Mater. Chem.* **22**, 12471 (2012).
6. Song, Q. *et al.* Quaternary Pseudocubic $Cu_2TMSnSe_4$ (TM = Mn, Fe, Co) Chalcopyrite Thermoelectric Materials. *Adv. Electron. Mater.* **2**, 1600312 (2016).
7. Li, M., Zhao, R. J., Su, Y. J., Hu, J., Yang, Z. & Zhang, Y. F. Hierarchically $CuInS_2$ Nanosheet-Constructed Nanowire Arrays for Photoelectrochemical Water Splitting. *Adv. Mater. Interfaces* **3**, 1600494 (2016).
8. Li, Q. *et al.* Growth of wurtzite $CuGaS_2$ nanoribbons and their photoelectrical properties. *J. Alloys Compd.* **567**, 127-133 (2013).
9. Yu, X., Shavel, A., An, X., Luo, Z., Ibanez, M. & Cabot, A. Cu_2ZnSnS_4-Pt and Cu_2ZnSnS_4-Au heterostructured nanoparticles for photocatalytic water splitting and pollutant degradation. *J. Am. Chem. Soc.* **136**, 9236-9239 (2014).
10. Ha, E., Lee, L. Y., Wang, J., Li, F., Wong, K. Y. & Tsang, S. C. Significant enhancement in photocatalytic reduction of water to hydrogen by Au/Cu_2ZnSnS_4 nanostructure. *Adv. Mater.* **26**, 3496-3500 (2014).
11. Yuan, M. *et al.* Cu_2ZnSnS_4-CdS heterostructured nanocrystals for enhanced photocatalytic hydrogen production. *Catal. Sci. Tech.* **7**, 3980-3984 (2017).
12. Chang, Z.-X. *et al.* High temperature recrystallization of kersterite Cu_2ZnSnS_4 towards

enhanced photocatalytic H_2 evolution. *Int. J. Hydrogen Energy* **40**, 13456-13462 (2015).

13. Ha, E. *et al.* Cu_2ZnSnS_4/MoS_2-Reduced Graphene Oxide Heterostructure: Nanoscale Interfacial Contact and Enhanced Photocatalytic Hydrogen Generation. *Sci. Rep.* **7**, 39411 (2017).
14. Chang, Z. X., Zhou, W. H., Kou, D. X., Zhou, Z. J. & Wu, S. X. Phase-dependent photocatalytic H_2 evolution of copper zinc tin sulfide under visible light. *Chem. Commun.* **50**, 12726-12729 (2014).
15. Chong, R., Wang, X., Chang, Z., Zhou, W. & Wu, S. SiO_2 loading combined with high temperature calcination of kesterite Cu_2ZnSnS_4 nanocrystals towards enhanced photocatalytic H_2 evolution. *Int. J. Hydrogen Energy* **42**, 20703-20710 (2017).
16. Yuan, M., Zhou, W.-H., Kou, D.-X., Zhou, Z.-J., Meng, Y.-N. & Wu, S.-X. Cu_2ZnSnS_4 decorated CdS nanorods for enhanced visible-light-driven photocatalytic hydrogen production. *Int. J. Hydrogen Energy* **43**, 20408-20416 (2018).
17. Li, Y. *et al.* Significant Enhancement of Hydrogen Production in MoS_2/Cu_2ZnSnS_4 Nanoparticles. *Part. Part. Sys. Charact.* **0**, 1700472 (2018).
18. Yu, X. *et al.* Cu_2ZnSnS_4–PtM (M = Co, Ni) Nanoheterostructures for Photocatalytic Hydrogen Evolution. *J. Phys. Chem. C* **119**, 21882-21888 (2015).
